# Thermal Transport and Functional Properties of Conformal Coatings for Automotive Electronics

**DOI:** 10.3390/ma19214699

**Published:** 2026-11-04

**Authors:** Wojciech Żyłka, Beata Giża, Małgorzata Pociask-Biały, Piotr Potera, Barbara Pilch-Pitera

**Affiliations:** 1 Faculty of Exact and Technical Sciences, Institute of Materials Engineering, University of Rzeszów, Pigonia 1, 35-310 Rzeszów, Poland; mpociask@ur.edu.pl (M.P.-B.);; 2 University of Rzeszów, 35-310 Rzeszów, Poland; 3 Faculty of Chemistry, Department of Polymers and Biopolymers, Rzeszów University of Technology, Powstańców Warszawy 6, 35-029 Rzeszów, Poland

**Keywords:** conformal coatings, automotive electronics, FTIR, UV–Vis–NIR, thermal conductivity, König pendulum hardness, protective coatings

## Abstract

This study presents a comparative analysis of the structural, electrical, optical, thermal, and mechanical properties of the conformal coatings used for electronic protection: acrylic coating, polyurethane coating, and silicone-modified acrylic coating. The chemical structure of the coatings was analyzed by Fourier-transform infrared (FTIR) spectroscopy, their electrical insulation properties were evaluated by electrical resistance measurements, and their optical properties were investigated by UV–Vis–NIR spectrophotometry over the 200–2500 nm range. The effects of coating type and actual coating thickness on heat transport and relative hardness were also evaluated. FTIR analysis revealed differences in the chemical structure of the investigated materials and confirmed the presence of functional groups characteristics of the respective coating types. The highest values electrical resistance recorded for the silicone-modified coating. UV–Vis–NIR measurements showed that both coating type and film thickness affected the reflectance characteristics in the visible and near-infrared ranges. The most favorable heat-transport parameters were obtained for selected acrylic and silicone-modified coating variants, whereas the thermal properties of the polyurethane coatings were more sensitive to changes in layer thickness. The obtained results confirm that the functional properties of conformal coatings depend on both their chemical structure and the actual thickness of the dry film, which should be taken into account when selecting an electrical insulating material when designing insulation in automotive electronic systems.

## 1. Introduction

The rapid development of the automotive industry has led to the widespread use of electronic systems responsible, among other functions, for powertrain control, safety systems, communication, and autonomous and driver-assistance functions [1]. Electronic components used in vehicles may operate under elevated humidity and in the presence of ionic contaminants, which can promote electrochemical migration and may lead to failures and short circuits. One method of limiting the effects of such factors is the application of conformal coatings to component surfaces and printed circuit boards [2]. Thermal management is also an important aspect of the design and protection of electronic systems. Thermal energy in devices can be transported by conduction, convection, and radiation, and the properties of the materials used affect the efficiency of these processes [3]. For materials and coatings used in electronics, radiative properties, including emissivity and the optical characteristics of the surface, are also important because they can influence radiative heat exchange [4]. Conformal coatings form a thin protective layer on the surface of electronic assemblies. Commonly used material groups include acrylic, polyurethane, silicone, epoxy, and parylene coatings [5]. However, the effectiveness of protection depends not only on the material type but also on surface preparation and coating application, because the application method can affect coating uniformity and the reliability of protected electronic assemblies [6]. The properties of conformal coatings are largely determined by the type of polymer matrix used [7]. Acrylic coatings are characterized by good moisture resistance, dielectric properties, and relatively easy application and removal during repair [8]. They are formed mainly by solvent evaporation, which facilitates subsequent removal but is also associated with limited resistance to some organic solvents [9]. Polyurethane coatings constitute another important group of materials used to protect electronic assemblies [7,9]. Studies conducted on populated printed circuit boards have shown that the behavior of such coatings under high-humidity conditions may depend, among other factors, on residues from the soldering process, component geometry, and the degree of surface coverage [10]. Silicone coatings are distinguished by high flexibility, moisture resistance, and favorable electrical-insulation properties, which are important in applications requiring high surface insulation resistance [11]. A comprehensive assessment of the suitability of conformal coatings for automotive electronics requires consideration of their chemical structure as well as their electrical, optical, thermal, and mechanical properties.

In automotive electronic systems, conformal coatings may be exposed to variable environmental conditions, including temperature fluctuations, elevated humidity, condensation, vibrations, and chemical contaminants, the severity of which depends on the location of the electronic components within the vehicle [12,13]. The climatic conditions relevant to assessing the reliability of automotive electrical and electronic equipment are specified in ISO 16750-4:2023 [13]. Studies on conformal coatings have demonstrated that moisture can penetrate the protective layer within a few hours, while the effectiveness of protection against corrosion and electrochemical migration depends largely on coating adhesion and the presence of solder flux residues [14]. It has also been confirmed that ionic contaminants introduced at various stages of printed circuit board manufacturing can significantly reduce surface insulation resistance and increase the risk of conductive dendrite formation under high-humidity conditions [15]. Furthermore, investigations of ceramic capacitors used in automotive electronics have shown that the presence of a conformal coating influences the progression of electrochemical migration under moisture exposure [12]. In automotive applications, the performance of coatings under cyclic temperature variations is also important, as such variations can induce stresses arising from differences in the coefficients of thermal expansion of the materials comprising the electronic assembly [16]. Therefore, the evaluation of coating properties under laboratory conditions represents a preliminary stage, whereas confirmation of their suitability for practical applications requires testing that accounts for the actual operating conditions of the protected electronic systems. In particular, it is necessary to verify the stability of electrical insulation properties under moisture exposure, resistance to thermal cycling, and coating performance on actual electronic assemblies.

Thermal management is an important aspect of the design and protection of electronic systems, particularly in the context of their miniaturization, increasing component packaging density, and operational reliability requirements [3]. Conformal coatings used in electronics primarily serve protective and electrical insulation functions, limiting the adverse effects of moisture and contaminants that may lead to electrochemical degradation and component failure [2,10]. At the same time, the application of an additional protective layer may affect heat transfer conditions in the protected electronic systems [17,18]. The potential of conformal coatings to improve heat dissipation from printed circuit boards has been demonstrated, among others, for composite materials containing graphene [18]. However, it should be noted that the incorporation of electrically conductive additives may also reduce the electrical resistivity of the material, which represents a significant limitation for electrically insulating coatings [19]. Therefore, the design of protective coatings for electronic applications requires consideration of both their thermal and electrical properties [2,10,19]. Heat transport in polymeric materials depends, among other factors, on their chemical structure and the nature of chemical bonds and interactions between polymer chains [17]. Differences in the chemical structure of coating materials may also be associated with variations in their functional properties [20,21]. Another important factor is the actual dry film thickness, which depends on the properties of the coating formulation and the conditions of the application and film formation processes [22].

Therefore, it is justified to evaluate the relationships between the chemical structure of these coatings, their dry film thickness, and selected electrical, optical, thermal, and mechanical properties.

In this context, the aim of the present study was to comparatively evaluate the electrical, optical, thermal, and mechanical properties of selected conformal coatings with different chemical structures, with particular emphasis on heat transfer through steel substrate–coating systems. Three types of coating materials were selected for investigation: an acrylic coating, a polyurethane coating, and a silicone-modified acrylic coating. The experimental program included identification of characteristic functional groups using Fourier-transform infrared (FTIR) spectroscopy, measurements of electrical insulation properties, analysis of UV–Vis–NIR spectral reflectance characteristics, evaluation of heat transfer through steel substrate–coating systems, and determination of pendulum hardness using the König method.

The study was designed to provide comparative laboratory data on the functional properties of selected conformal coatings and to assess potential relationships between their chemical structure, dry film thickness, and the electrical, reflectance, and thermal properties of the investigated systems. The results were considered in the context of potential applications in automotive electronics.

## 2. Materials and Methods

### 2.1. Research Materials

Three conformal coating materials intended for the protection of electronic systems were used in this study: 419D Acrylic Conformal Coating (MG Chemicals, Burlington, ON, Canada), 4223F Premium Polyurethane Conformal Coating (MG Chemicals, Burlington, ON, Canada) and 422B Silicone Modified Conformal Coating (MG Chemicals, Burlington, ON, Canada) [23]. The selected materials enabled comparison of three different protective coating systems used in electronics: acrylic, polyurethane, and silicone-modified acrylic [7].

The main characteristics of the investigated coating materials, as specified in the manufacturer’s technical documentation [23], are described below.

The acrylic coating (AC) forms a protective layer through physical solvent evaporation. Its dust-drying time is approximately 10 min at 22 °C, while full drying requires 24 h at room temperature or 10 min at 100 °C. The coating is colorless, transparent, and smooth, and is intended for the protection of printed circuit boards (PCBs) and electronic components.

The polyurethane coating (PU) forms a protective layer through thermal curing at elevated temperature. The drying time is approximately 2 h at 100 °C. The coating is colorless, transparent, and smooth, and is intended for the protection of PCBs and electronic components operating in harsh environments.

The silicone-modified acrylic coating (SIL) forms a protective layer through physical solvent evaporation. Its dust-drying time is approximately 5–7 min at room temperature. The coating is colorless, transparent, and smooth, and is intended for the protection of PCBs operating under high temperature and high humidity.

### 2.2. Preparation of Coated Samples

Two types of substrates were used to prepare the specimens: TQC contrast cards with black-and-white fields and smooth metal test panels. Before coating application, all substrates were cleaned and degreased to remove surface contamination and ensure reproducible application conditions. The coatings were applied using a TestAn model 219 automatic film applicator (Agencja Anticorr Gdańsk Sp. z o.o., Gdańsk, Poland). Specimens were prepared using applicator gaps corresponding to nominal wet-film thicknesses of 30, 60, and 90 µm. During application, the device speed was set to a linear travel speed of 8 mm/s. After application, the specimens were left on a flat surface to allow solvent evaporation and formation of a continuous polymer layer. After drying, the actual dry-film thickness was determined by measurements performed at several locations on each specimen. The mean measured dry-film thickness was used in the subsequent analysis of coating properties. It was lower than the nominal wet-film thickness because of solvent evaporation and differences in film-formation behavior [22,24].

### 2.3. Dry Film Thickness

The thickness of the dry film was measured with micro-TRI-Gloss-µ tester according to ISO 2808 [25]. 10 thickness measurements were made for each sample. Values are presented as mean ± standard deviation (SD) of dry-film thickness measurements.

### 2.4. FTIR Spectroscopy

The structural analysis of the investigated coatings was carried out using Fourier-transform infrared (FTIR) spectroscopy with a Thermo Scientific Nicolet 6700 FT-IR spectrophotometer (Thermo Fisher Scientific, Waltham, MA, USA) equipped with a helium–neon (HeNe) laser. Spectra were recorded in the range of 600–4000 cm^−1^ at a resolution of 4 cm^−1^. This method enables the identification of functional groups present in the investigated materials and assessment of coating chemical structure based on characteristic absorption bands [26]. Infrared spectroscopy examines the interaction of IR radiation with matter, in which absorption of radiation causes excitation of chemical-bond vibrations in molecules [27]. Spectral interpretation was performed in the mid-infrared region, where the fundamental stretching and bending vibrations of most functional groups in organic and inorganic compounds occur [26,28]. FTIR spectra were used to identify characteristic bands corresponding to structural components of the acrylic, polyurethane, and silicone-modified coatings. This analysis enabled assessment of the consistency of the spectra with the declared polymer base of the investigated coatings [29].

### 2.5. Polarized Optical Microscopy (POM) Measurements

A VHX-7000 polarized optical microscope with an EA-300 laser by Keyence (Osaka, Japan) was employed for this study. The morphology of each sample was assessed in transmitted light.

### 2.6. UV–Vis–NIR Spectrophotometry

The optical properties of the investigated coatings were determined by UV–Vis–NIR spectrophotometry with an integrating sphere. This method is based on analysis of changes in radiation intensity after transmission through a material or reflection from its surface, while the energy of electromagnetic radiation depends on wavelength according to the Planck–Einstein relation [30]. In the UV–Vis range, radiation may induce electronic transitions, whereas in the NIR range the recorded features are mainly overtones and combination bands of functional-group vibrations such as C–H, O–H, and N–H [31]. Measurements were performed using an Agilent Cary 5000 UV–Vis–NIR spectrophotometer (Agilent Technologies, Santa Clara, CA, USA). The reflectance measurement range covered wavelengths from 200 to 2500 nm, including ultraviolet radiation, visible light, and near-infrared radiation. The measuring accuracy of the spectrometer Cary 5000 for the wavelength in the UV–Vis range is Δλ = ±0.08 nm, while in the case of the reflectance the accuracy is ΔR = 0.07%. For the NIR, the wavelength measurement error is 0.4 nm, and for the reflection coefficient 0.2%. An uncoated reference specimen and specimens coated with the investigated protective coatings at different thicknesses were analyzed. For coatings applied to opaque substrates, reflectance measurements were used as a non-destructive method for evaluating the optical properties of multilayer systems [32,33]. The interpretation of the results considered the nature of radiation reflected from the surface and from within the coating layer. For quantitative interpretation of reflectance spectra, the Kubelka–Munk function can be used to transform a reflectance spectrum into a form related to material absorption [34,35]. The optical characteristics were also considered in the context of previous studies on the transmittance of industrial coatings [36].

### 2.7. Thermal Measurements and Calculations

Thermal-property measurements were performed to determine the effect of protective coating type on heat transport through the substrate–coating system. Interpretation of the results was based on Fourier’s law, according to which heat flux density is proportional to the temperature gradient in a material [37,38], representing the fundamental mechanism of energy transport in solids. [37,39].

**Figure 1:**
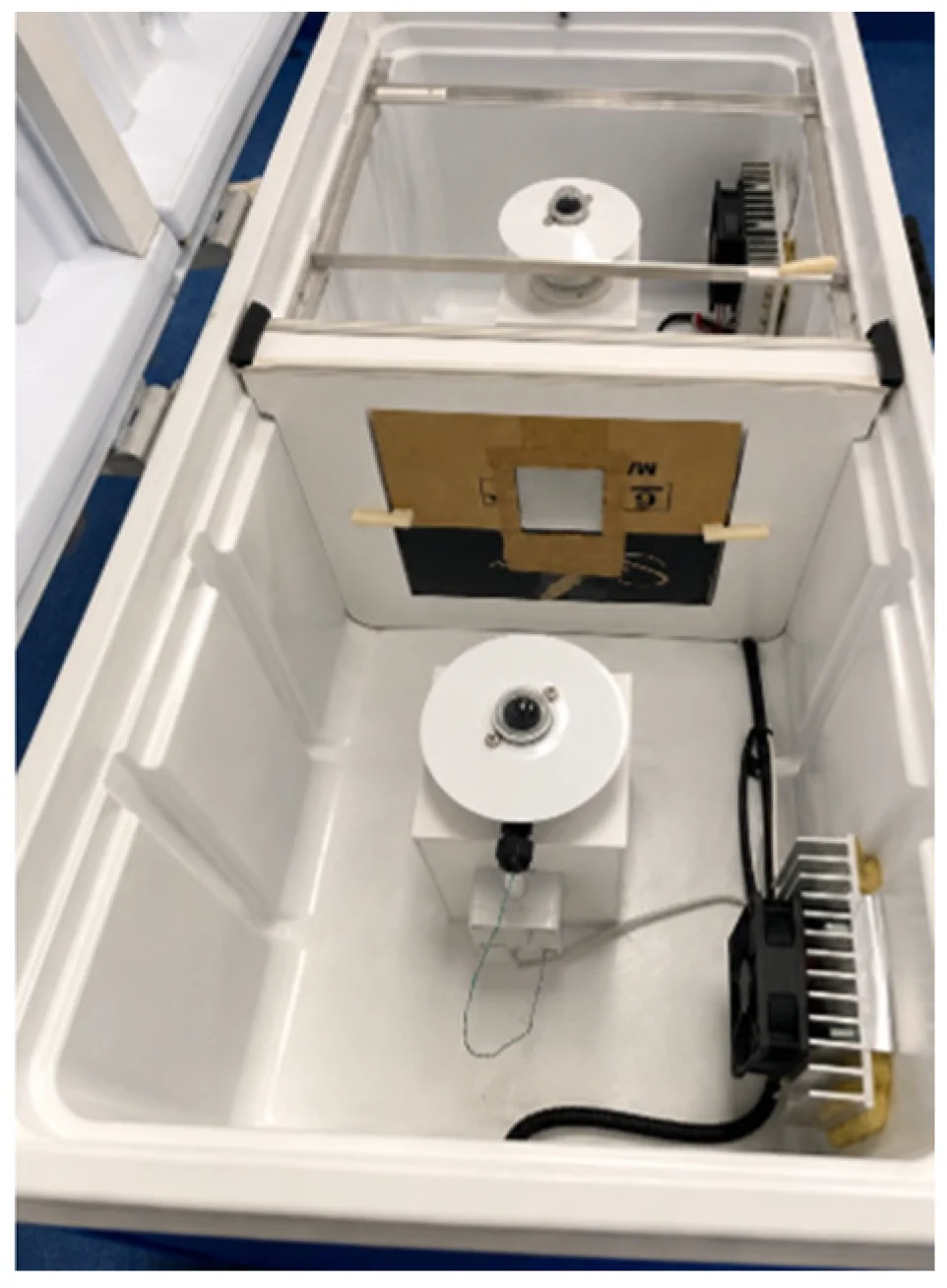
Test stand for thermal properties.

Measurements were conducted using a laboratory heat-transport test stand located in the Renewable Energy Laboratory at the University of Rzeszow (Figure 1, University of Rzeszow, Rzeszow, Poland). The setup consisted of two insulated chambers: a heating chamber and a cooling chamber, each with a volume of V = 0.07488 m^3^ (with accuracy 0.0000001 m^3^). The specimen was mounted in an opening between the chambers. The total area of the opening was A = 0.0506 m^2^ (*u*_A_ = 0.000004 m^2^), while the exposed specimen area was maintained at B = 0.0059 m^2^ (*u*_B_ = 0.000001 m^2^) using a paper-based insulating tape frame. The remaining area was occupied by a (5.50 ± 0.05) mm thick cardboard holder d_cboard_.

Temperatures on both sides of the specimen were recorded at 2 s intervals using thermocouples (±0.01 °C accuracy). Each run started at ambient temperature; one chamber remained at room temperature while the other was heated to approximately 42 °C. The air density variation with temperature was taken into account. Measurements on coated steel substrates were performed in duplicate and averaged.

Thermal properties were determined by analyzing the relationship between heat flow rate (Q) and temperature difference between chambers (ΔT = T_heat_ − T_cool_). The thermophysical properties’ temperature dependence was incorporated into the calculations [38,40,41]. To ensure accuracy, the analysis focused exclusively on the linear region after reaching a quasi-steady state (at ΔT ≈ 1 °C).

One-dimensional, steady-state heat conduction through a flat layer is described by Fourier’s law: $Q=\lambda \left(\frac{S}{d}\right)\Delta T=a\;\Delta T$, where Q—heat flow rate (thermal power) passing through the sample [W or J/s], λ—thermal conductivity of the material [W/m·K], S—heat transfer area (cross-sectional area of the sample) [m^2^], d—sample thickness (steel substrate + coating) [m], ΔT—temperature difference between chambers (T_heat_ − T_cool_) [K or °C], *a*—slope of the linear fit (*a* = λ S/d = U S, [W/K], where U is the heat transfer coefficient in W/m^2^·K).

The slope a determined from the linear fit served as the key parameter for calculating the overall heat transfer coefficient (U), thermal conductivity (λ), and thermal resistance (R). Uncertainty in slope a was evaluated as the maximum deviation from the average and propagated according to standard error propagation principles. The mean effective thickness of the specimen–holder assembly, d*_sh_*, weighted by the respective area fractions, was calculated as according to Equation (1):

(1) $$d_{\mathrm{sh}}=\frac{\left(A-B\right)\cdot d_{\mathrm{cboard}}+B\cdot d_{\mathrm{steel}+\mathrm{coating}}}{A}$$

The overall heat transfer coefficient of the system, U_sh_, representing two parallel heat-flow paths through the cardboard holder and specimen, is given by Equation (2) [37,38,40]:

(2) $$U_{\mathrm{sh}}=\frac{\left(A-B\right)\cdot U_{\mathrm{cboard}}+B\cdot U_{\mathrm{steel}+\mathrm{coating}}}{A}.$$

Substituting the relationships for the linear slopes into Equation (2) yields Equation (3), where the term $\frac{A-B}{A}$ accounts for the effective fraction of the cardboard holder area during measurement:

(3) $$\frac{\lambda_{\mathrm{steel}+\mathrm{coating}}}{d_{\mathrm{steel}+\mathrm{coating}}}=\frac{1}{B}\left(a_{\mathrm{sh}}-a_{\mathrm{cboard}}\frac{A-B}{A}\right)=\frac{a_{\mathrm{sh}-}a_{\mathrm{cboard}}}{B}+\frac{a_{\mathrm{cboard}}}{A},$$

where *a*_sh_ denotes the slope for the sample–holder assembly, and *a*_cboard_ represents the slope for the cardboard holder alone (*a*_cboard,mean_ ± $u_{a_{\mathrm{cboard}}}$= 0.1023 ± 0.0005 [W/K]).

Applying the general law of uncertainty propagation [41] for *U*_steel+coating_ in Equation (4):

(4) $$u^{2}\left(U_{\mathrm{steel}+\mathrm{coating}}\right)=\sum_{i}{\left(\frac{\partial U_{\mathrm{steel}+\mathrm{coating}}}{\partial x_{i}}\right)}^{2}u^{2}\left(x_{i}\right)$$

leads to standard uncertainty expression in Equation (5):

(5) $$u\left(\frac{\lambda_{\mathrm{steel}+\mathrm{coating}}}{d_{\mathrm{steel}+\mathrm{coating}}}\right)=\sqrt{{\left(\frac{u_{a_{\mathrm{sh}}}}{B}\right)}^{2}+{\left(\frac{A-B}{A\cdot B}u_{a_{\mathrm{cboard}}}\right)}^{2}+{\left(\frac{a_{\mathrm{cboard}}}{A^{2}}u_{A}\right)}^{2}+{\left(\frac{a_{\mathrm{sh}}-a_{\mathrm{cboard}}}{B^{2}}u_{B}\right)}^{2}}$$

where $u_{a_{\mathrm{sh}}}$ and $u_{a_{\mathrm{cboard}}}$ are the uncertainties of the respective slopes, and *u*_A_ and *u*_B_ are the area measurement uncertainties.

The standard uncertainty of thermal conductivity $u_{\lambda_{\mathrm{steel}+\mathrm{coating}}}$ was evaluated as Equation (6):

(6) $$u_{\lambda_{\mathrm{steel}+\mathrm{coating}}}=\sqrt{{\left(d_{\mathrm{steel}+\mathrm{coating}}\cdot u_{U_{\mathrm{steel}+\mathrm{coating}}}\right)}^{2}+{\left(U_{\mathrm{steel}+\mathrm{coating}}\cdot u_{d_{\mathrm{steel}+\mathrm{coating}}}\right)}^{2}}$$

where $u_{d_{\mathrm{steel}+\mathrm{coating}}}$ denotes the specimen thickness uncertainty.

The standard uncertainty of thermal resistance $u_{R_{\mathrm{steel}+\mathrm{coating}}}$ was determined from the reciprocal relation in Equation (7):

(7) $$u_{R_{\mathrm{steel}+\mathrm{coating}}}=\frac{u_{U_{\mathrm{steel}+\mathrm{coating}}}}{{\left(U_{\mathrm{steel}+\mathrm{coating}}\right)}^{2}}.$$

Since *u*_A_ ≅ *u*_B_ = 0.000004 m^2^ in the estimation of in Formula (6) and in the estimation of in Formula (7), the $u_{d_{\mathrm{steel}+\mathrm{coating}}}$ = 0.00001 m, only the first term of the sum under the square root sign should be retained.

The standard uncertainty $u_{d_{sh}}$ was evaluated via partial derivatives with respect to d_cboard_, d_steel+coating_, A, and B. Tables 2–4, in the Section 3.5 present the calculated d_sh_ values alongside the conservative maximum uncertainty, $\left(u_{d_{\mathrm{sh}}}\right)$max.

### 2.8. Surface Resistivity Measurements

Surface resistivity measurements were performed to compare the insulating properties of the investigated coatings. The tests were carried out using a Keithley 6517B electrometer and Keithley 8009 Resistivity Test Fixture with cylindrical electrodes (Keithley Instruments, Cleveland, OH, USA) at a test voltage of 400 V.

Square samples with a length of 90 mm of the conditioned coatings at 25 °C and 45% relative humidity for 7 days were used for the surface resistivity test. A “Forced Voltage Measured Current” measurement method was implemented: during the experiments, voltage *U* was forced along the sample’s surface and the electric current *I* was measured. The applied voltage and measured current were used then for surface resistance calculation, as follows, in Equation (8):

(8) $$R_{s}=\frac{U}{I}$$

For each sample, two series of measurements were performed, and two readings were taken in each series.

Measurements were performed on 2 samples of each type, for each sample, 25 measurements of the voltage and the electric current were performed in the repeatability conditions (charging duration 1 min, temp. 25 °C, relative humidity 45%) and then the average value of R_s_ was calculated. On the basis of R_s_, the average value of the surface resistivity ρ_s_ was calculated, according to Equation (9):

ρ*_s_* = K_s_ R_s_, (9)

where K_S_ is the cell’s construction constant.

The constant depends on the cylindrical electrodes effective perimeter *l* and the distance *g* between both electrodes in Equation (10):

(10) $$K_{s}=\frac{l}{g}.$$

For the Keithley’s cell, the constant equals to 53.4.

### 2.9. Pendulum Hardness Measurements

The mechanical resistance of the investigated coatings was evaluated using the König pendulum hardness method. This method is used to assess coating hardness based on the damping of pendulum oscillations while the pendulum rests on the surface of the tested layer [42]. Harder coatings cause slower damping of oscillations, whereas softer coatings lead to a faster decay of pendulum motion. Measurements were carried out using a König pendulum hardness tester (BYK-Gardner, Geretsried, Germany). Before testing, the specimen was placed on the measurement table and stabilized to prevent movement during the test. The pendulum was then lowered so that its spherical supports rested on the coating surface, after which oscillatory motion was initiated. The instrument recorded the number of oscillations, which was used as the basis for determining coating hardness. Three measurements were performed for each specimen under the same laboratory conditions, and the mean value was calculated. Before testing the coated specimens, a reference-glass measurement was performed, yielding 170 oscillations and confirming correct instrument operation and measurement procedure.

## 3. Results and Discussion

### 3.1. Thickness of Dry Coatings

Actual dry-film thicknesses were lower than the nominal wet-film values (30, 60, and 90 µm) due to the rapid evaporation of volatile components, formulation-dependent rheological properties, and differences in film-formation behavior, the final dry-film thickness was not always proportional to the nominal applicator setting.

The investigated specimens were designated according to their coating formulation as AC-1–AC-3 (acrylic), PU-1–PU-3 (polyurethane), and SIL-1–SIL-3 (silicone-modified acrylic). Within each material group, the specimens were arranged in ascending order of the experimentally measured dry-film thickness. The mean measured dry-film thickness values are summarized in Table 1.

In the specimen labels used throughout the Results section, the values 30, 60, and 90 µm refer to the nominal wet-film thickness, whereas quantitative analyses were based on the measured mean dry-film thickness. The measured dry-film thickness varied between coating systems and applicator settings, supporting the use of the measured mean dry-film thickness in subsequent quantitative analyses.

### 3.2. FTIR Analysis

#### 3.2.1. FTIR Spectrum Analysis of the Acrylic Coating

**Figure 2:**
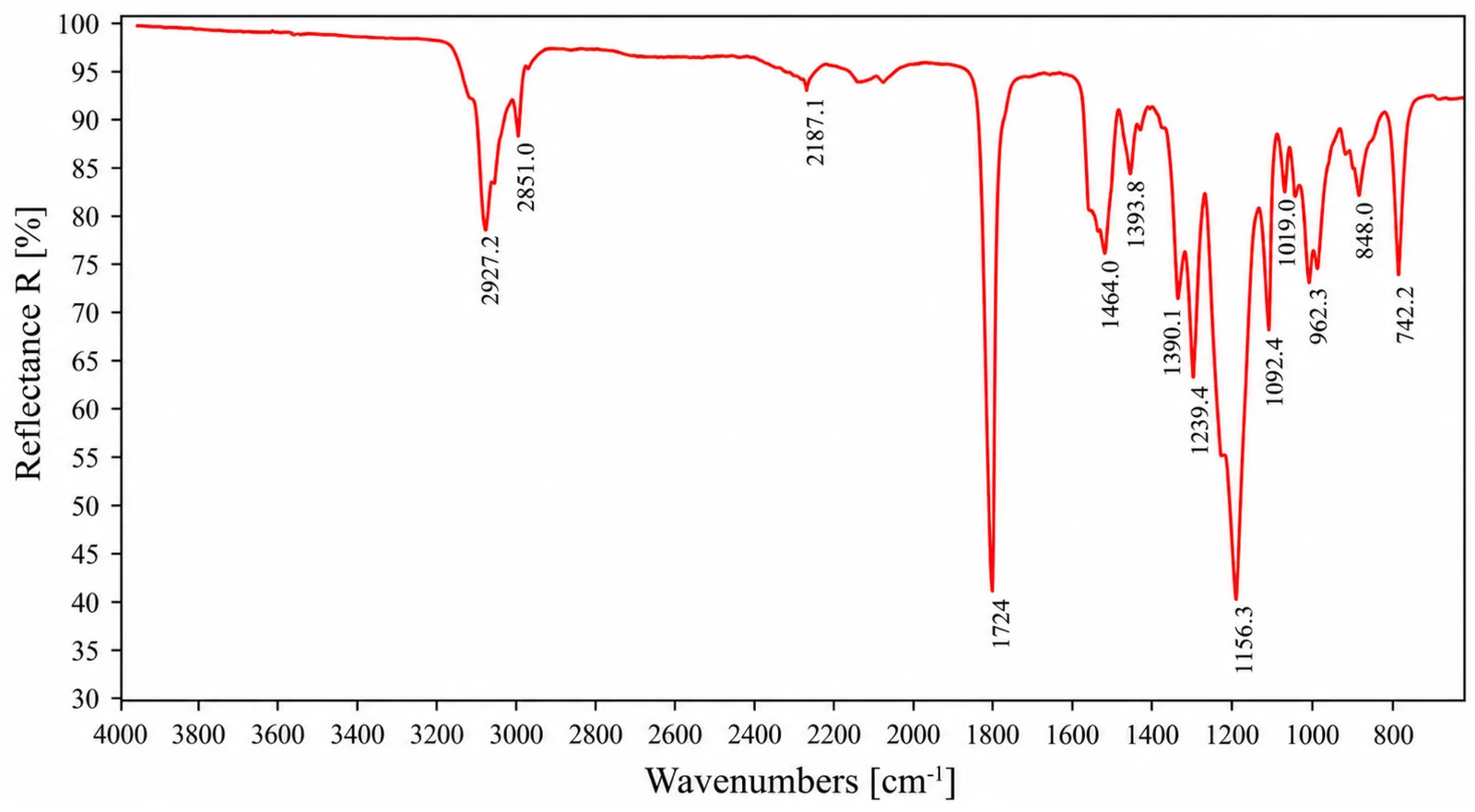
FTIR spectrum of the acrylic coating.

**Figure 3:**
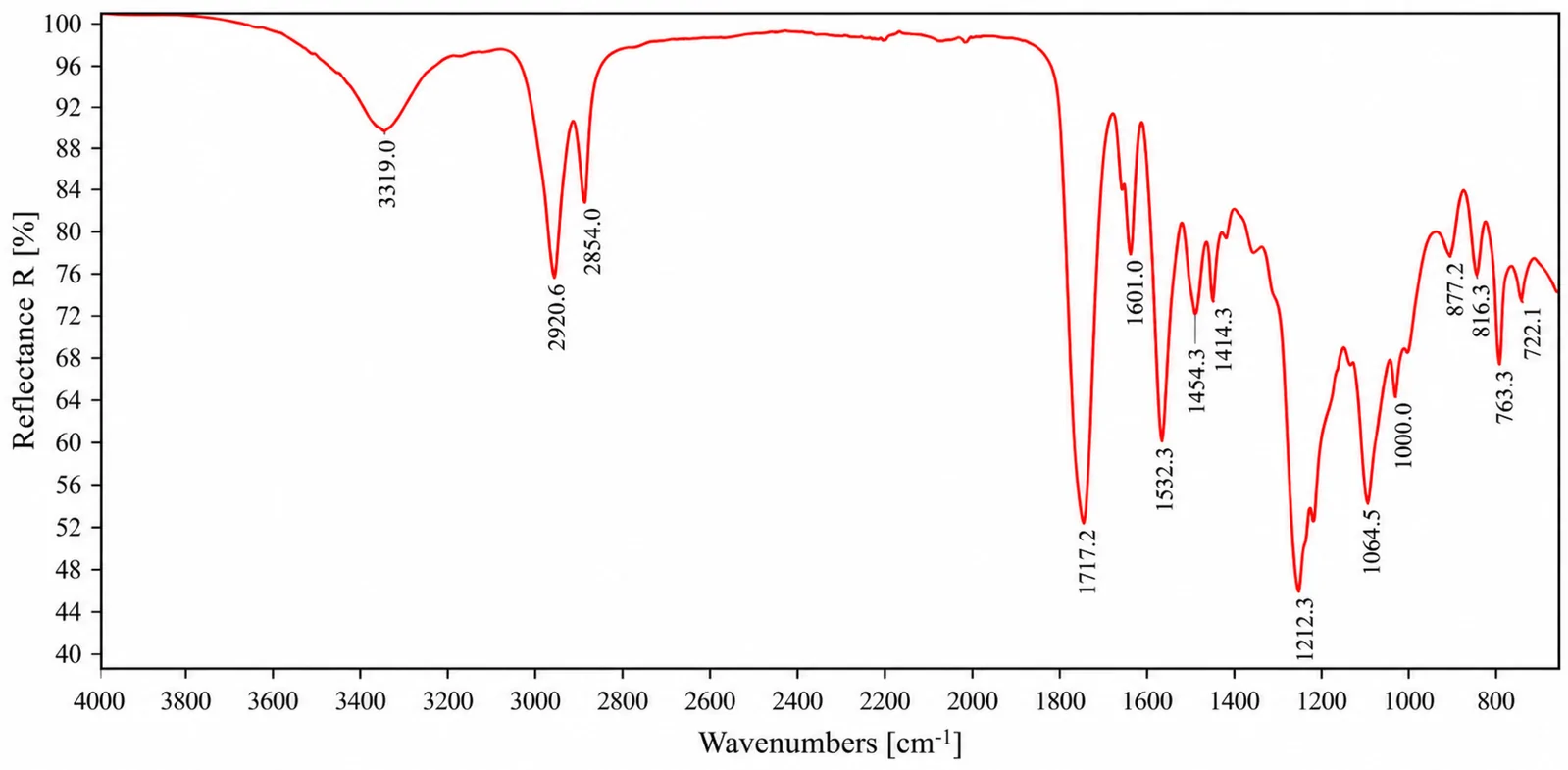
FTIR spectrum of the polyurethane coating.

**Figure 4:**
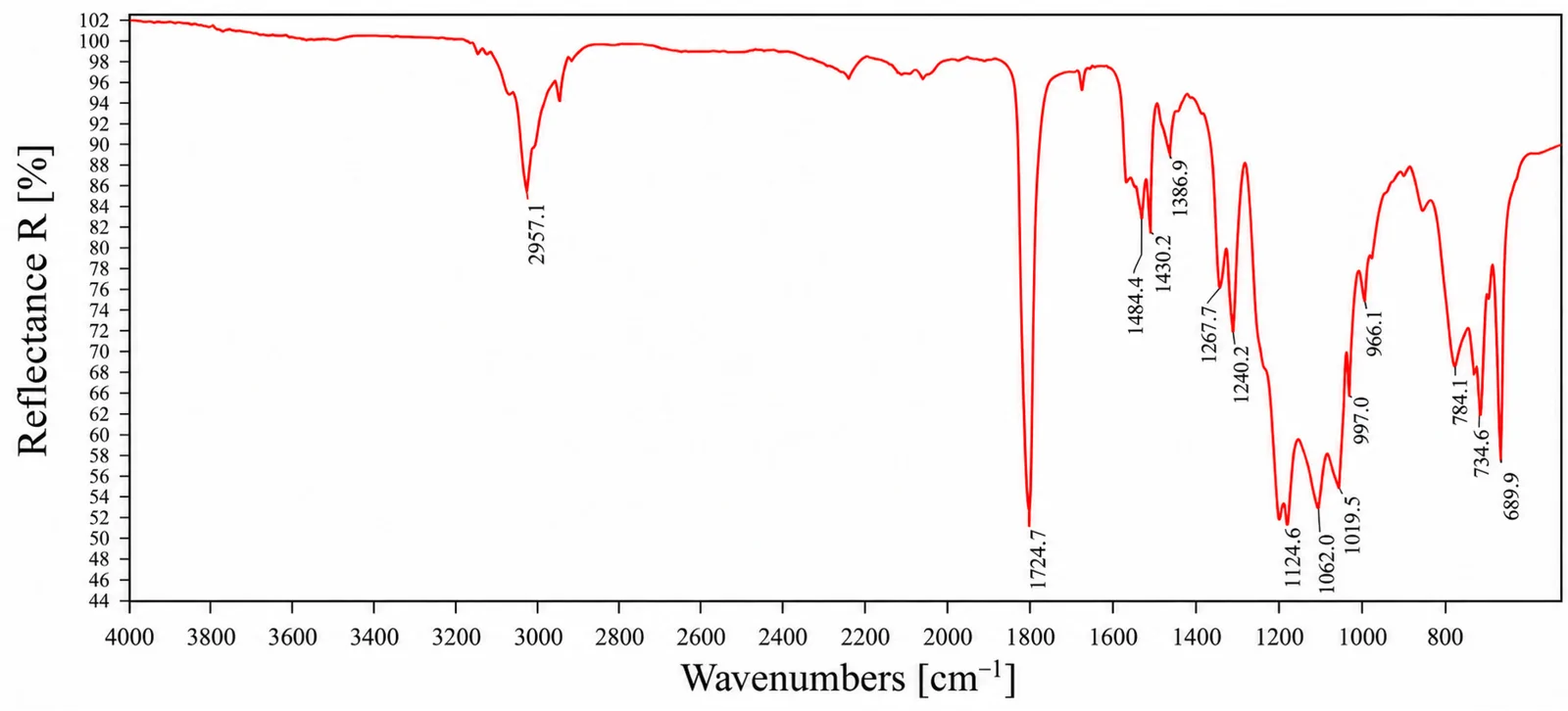
FTIR spectrum of the silicone modified acrylic coating.

FTIR spectroscopy was used to identify characteristic functional groups present in the tested coatings and to assess the consistency of the observed absorption bands with the declared chemical structure of these materials. The recorded FTIR spectra of the coatings are presented in Figure 2, Figure 3 and Figure 4.

The recorded FTIR spectrum of the acrylic coating (Figure 2) reveals a series of signals indicating the presence of structures typical of acrylic polymers.

The most intense band, appearing at a wavenumber of approximately 1723 cm^−1^, corresponds to the stretching vibrations of the carbonyl group (C=O) found in ester moieties attached as substituents to the acrylic resin backbone. This assignment is consistent with FTIR studies of acrylate-based polymers, in which a strong band near 1723–1725 cm^−1^ is characteristic of ester carbonyl stretching vibrations [43]. Bands arising from the stretching vibrations of aliphatic carbon–hydrogen (C–H) bonds are visible in the regions around 2927.2 cm^−1^ and 2851.0 cm^−1^. The intense bands in the 1012–1239 cm^−1^ range can be associated with C–O and C–O–C stretching vibrations of ester groups [44]. In summary, the FTIR spectrum of the acrylic coating revealed characteristic absorption bands associated with ester and aliphatic functional groups typical of acrylic polymers. The identified groups confirm consistency with the declared acrylic structure.

#### 3.2.2. FTIR Spectrum Analysis of the Polyurethane Coating

The FTIR spectrum of the polyurethane coating (Figure 3) exhibited a characteristic broad band at a wavenumber of 3319.0 cm^−1^, corresponding to the stretching vibrations of N–H groups. Bands associated with the asymmetric and symmetric stretching vibrations of C–H bonds in the methyl and methylene groups of the polyurethane chain’s aliphatic segments are present at approximately 2920.6 cm^−1^ and 2854.0 cm^−1^. The strongest band appears at a wavenumber of approximately 1717.2 cm^−1^ (the so-called amide I band) and arises from the stretching vibrations of carbonyl groups (C=O), forming urethane moieties [45]. The band at 1601.0 cm^−1^ originates from the stretching vibrations of the isocyanate aromatic ring. The band at 1532.3 cm^−1^ is associated with the deformation vibrations of N–H groups (the so-called amide II band) [46]. Intense bands at wavenumbers of approximately 1221.3 cm^−1^, 1064.5 cm^−1^, and 1000.0 cm^−1^ are associated with the presence of C–O and C–O–C bonds, forming urethane and ester moieties derived from the polyol components present in the polyurethane structure. At 816 cm^−1^, there is a band characteristic of deformation vibrations outside the C–H plane of aromatic rings substituted in the 1,2,4- position, originating from toluylene 2,4-diisocyanate [47].

The absence of the isocyanate –N=C=O band near 2268 cm^−1^ indicates complete reaction during coating curing and confirms a properly formed polyurethane structure.

In summary, the FTIR spectrum of the polyurethane coating revealed characteristic absorption bands derived from polyurethane containing soft segments composed of aliphatic polyester and hard segments composed of 2,4-toluene diisocyanate. Overall, the identified absorption bands are consistent with the expected chemical structure of the polyurethane coating.

#### 3.2.3. FTIR Spectrum Analysis of the Silicone Coating

The FTIR spectrum of the silicone-modified acrylic coating showed bands originating from polysiloxane chain segments (Figure 4). An intense band associated with the stretching vibrations of the Si–O–Si bonds occurs at approximately 1019.5 cm^−1^ [48]. The high bond dissociation energy of the Si–O bonds in the siloxane backbone (up to approximately 460 kJ mol^−1^) compared with C–O bonds (approximately 345 kJ mol^−1^) contributes to the excellent thermal stability of polysiloxanes. The chemical stability of the Si–O–Si network additionally provides resistance to thermal and chemical degradation, making polysiloxane-based coatings attractive for protective applications in electronics operating at elevated temperatures [49]. Also present are bands in the range of approximately 1267.1 cm^−1^, attributed to the bending vibrations of the Si–CH_3_ groups attached to silicone chains, which, due to their hydrophobic nature, provide the coatings with high water resistance. At 784.1 cm^−1^, band associated with the rocking vibrations of the -CH_3_ groups attached to the silicon atoms is present [50].

At 1700 cm^−1^, a band characteristic of the stretching vibrations of the carbonyl groups (C=O), which are present in the structure of acrylates, is also present. At approximately 2967.1 cm^−1^, a band associated with the stretching vibrations of the C–H bonds occurring in the methyl groups attached to the silicon atoms was observed.

In summary, the FTIR spectrum of the silicone-modified acrylic coating revealed characteristic absorption bands associated with both polysiloxane and acrylic structures. Overall, the identified bands are consistent with the expected chemical structure of the silicone-modified acrylic coating, combining characteristic features of polysiloxane and acrylic components.

Overall, FTIR spectroscopy confirmed the presence of characteristic functional groups in all investigated coatings and allowed the identification of structural elements such as aliphatic chains and aromatic rings, which can significantly influence the morphology and properties of the coatings.

### 3.3. Polarized Optical Microscopy Analysis

**Figure 5:**
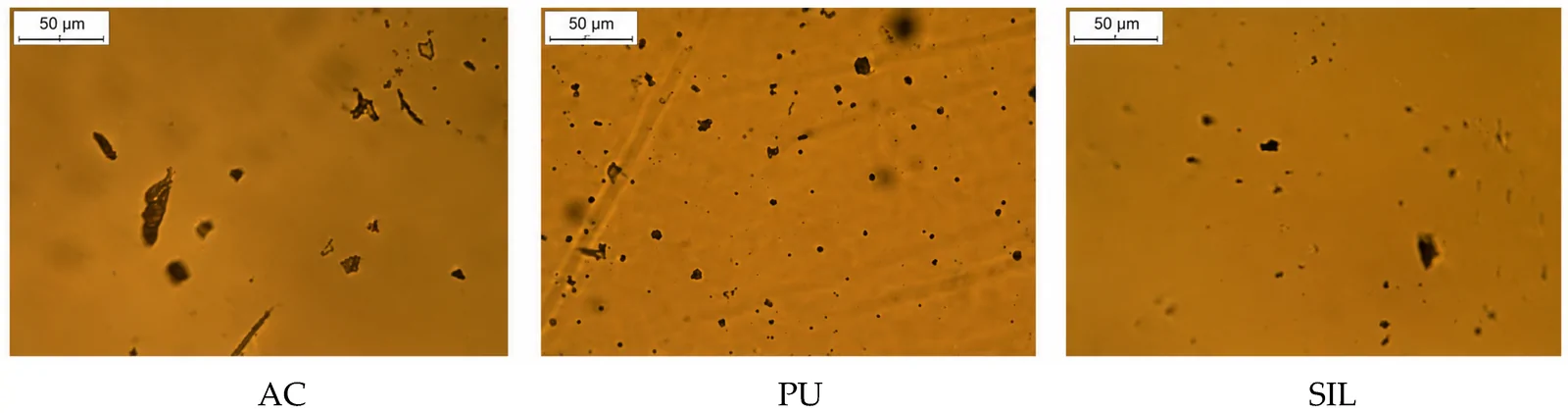
Microscopic images of the dry coatings in non-polarized light.

To examine the morphology of the dry coatings, they were observed using a polarizing optical microscope in transmitted light. Observations were performed in both unpolarized and polarized light. The microscopic images of the dry coatings in unpolarized light are shown in Figure 5.

Microscopic images obtained under unpolarized light indicate that the investigated coatings are characterized by a transparent matrix containing a small number of dispersed structures. In the acrylic coating, fewer structures were observed; however, they were larger in size. Smaller structures were present in the silicone coating, whereas the smallest features were observed in the polyurethane coating.

**Figure 6:**
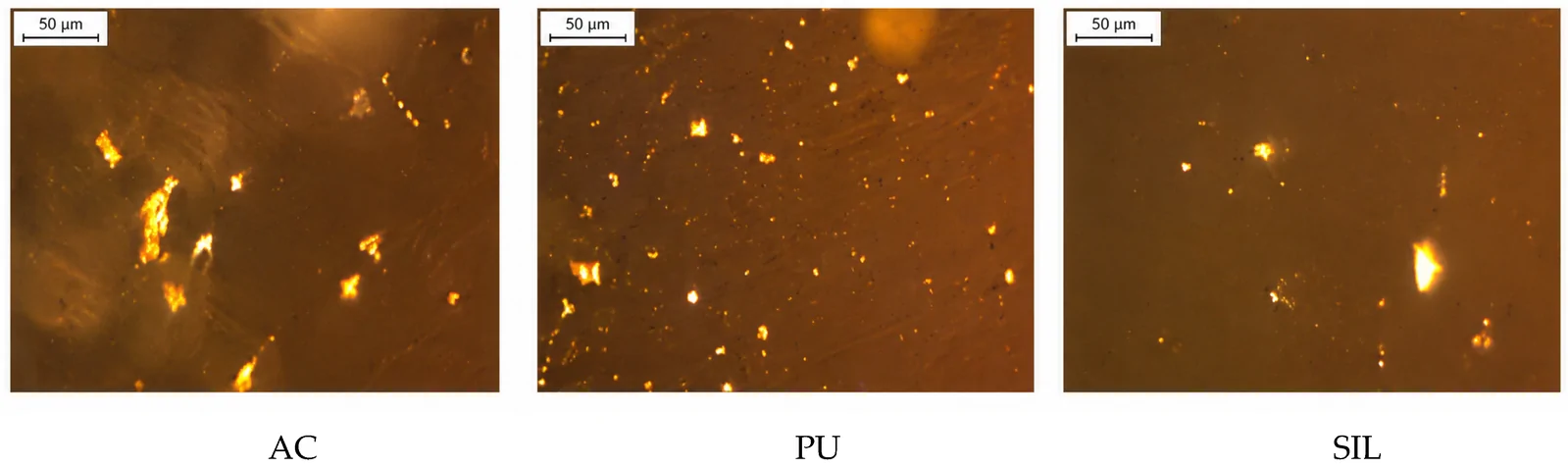
Microscopic images of the dry coatings in polarized light.

To characterize the morphology of the observed structures in greater detail, additional observations were performed under polarized light (Figure 6).

As shown in Figure 6, some of these structures exhibit enhanced optical contrast under these conditions. This phenomenon may be associated with the optical birefringence of the structures, which is used in polarized light microscopy to characterize the organization and morphology of crystalline structures in polymers [51].

One possible interpretation of the observed structures is that they represent regions with crystalline order associated with the organization of polymer chain segments. The influence of such structures on the optical properties of coatings depends, among other factors, on their size, volume fraction, and the differences in refractive indices between the individual regions of the material [52].

### 3.4. Spectrophotometric Analysis of the Reflectance Properties of the Coatings

**Figure 7:**
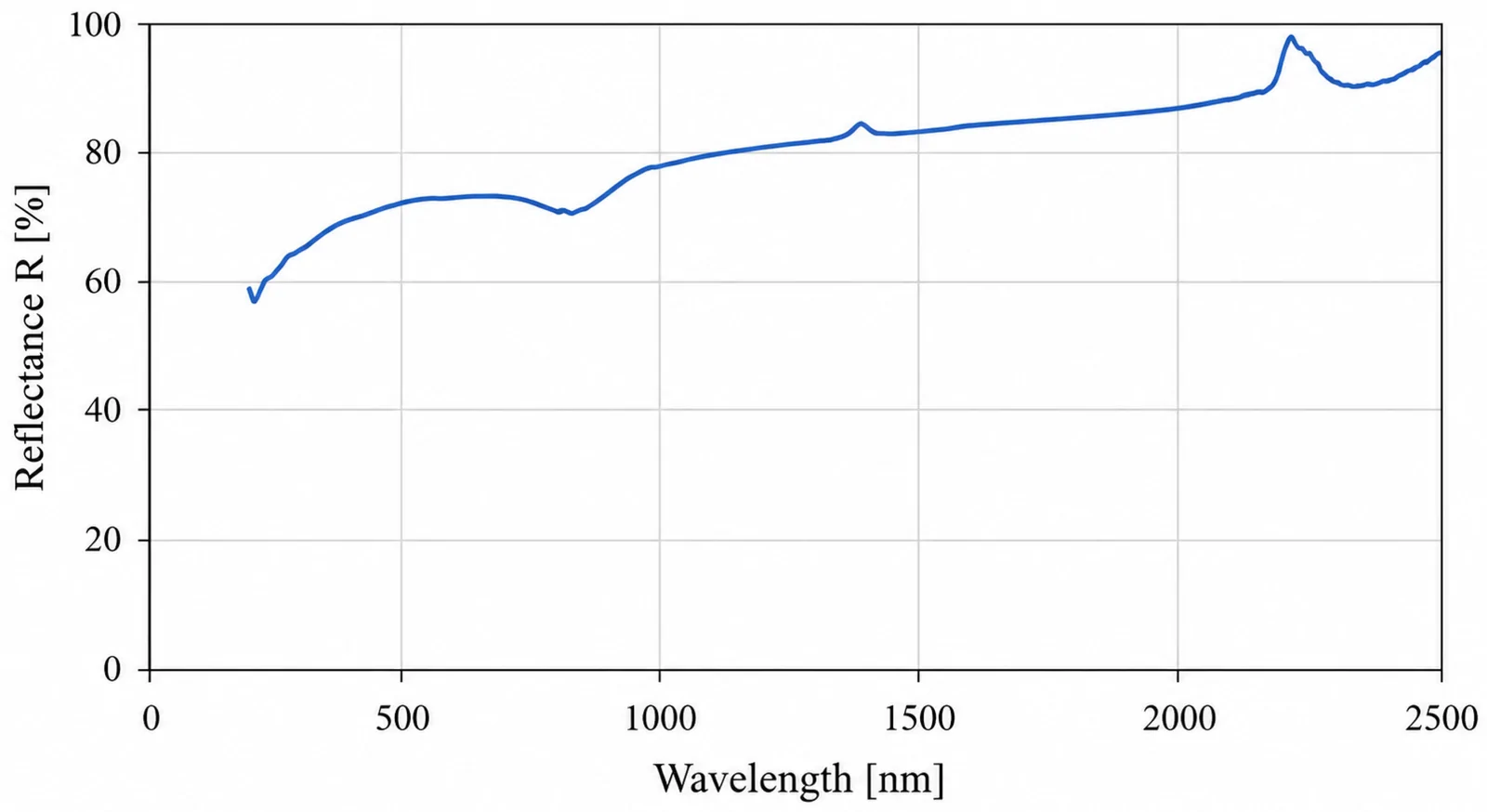
Reflectance spectrum of the uncoated reference panel.

**Figure 8:**
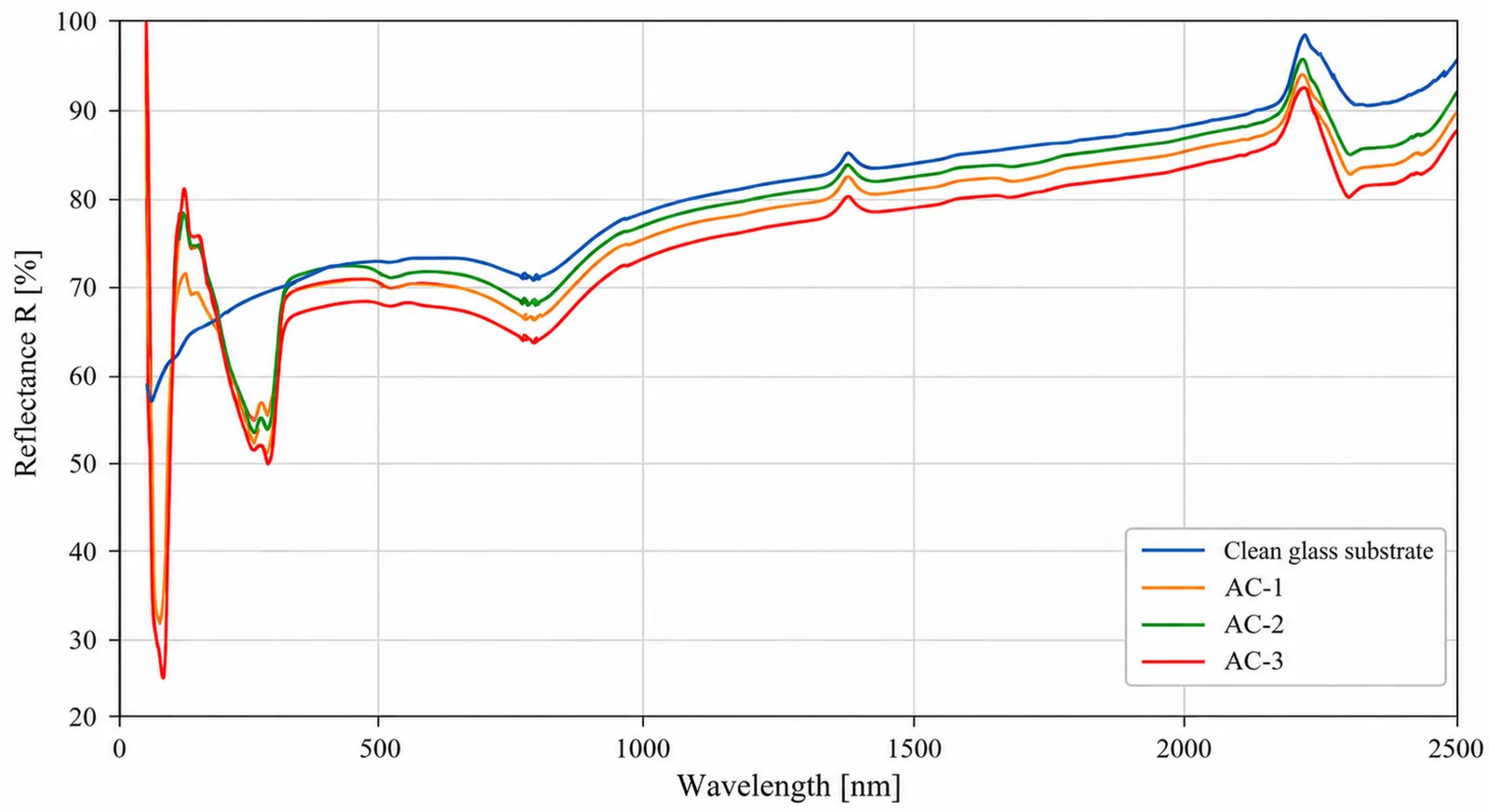
Comparison of reflectance spectra of the uncoated reference panel and acrylic-coated specimens AC-1, AC-2 and AC-3.

**Figure 9:**
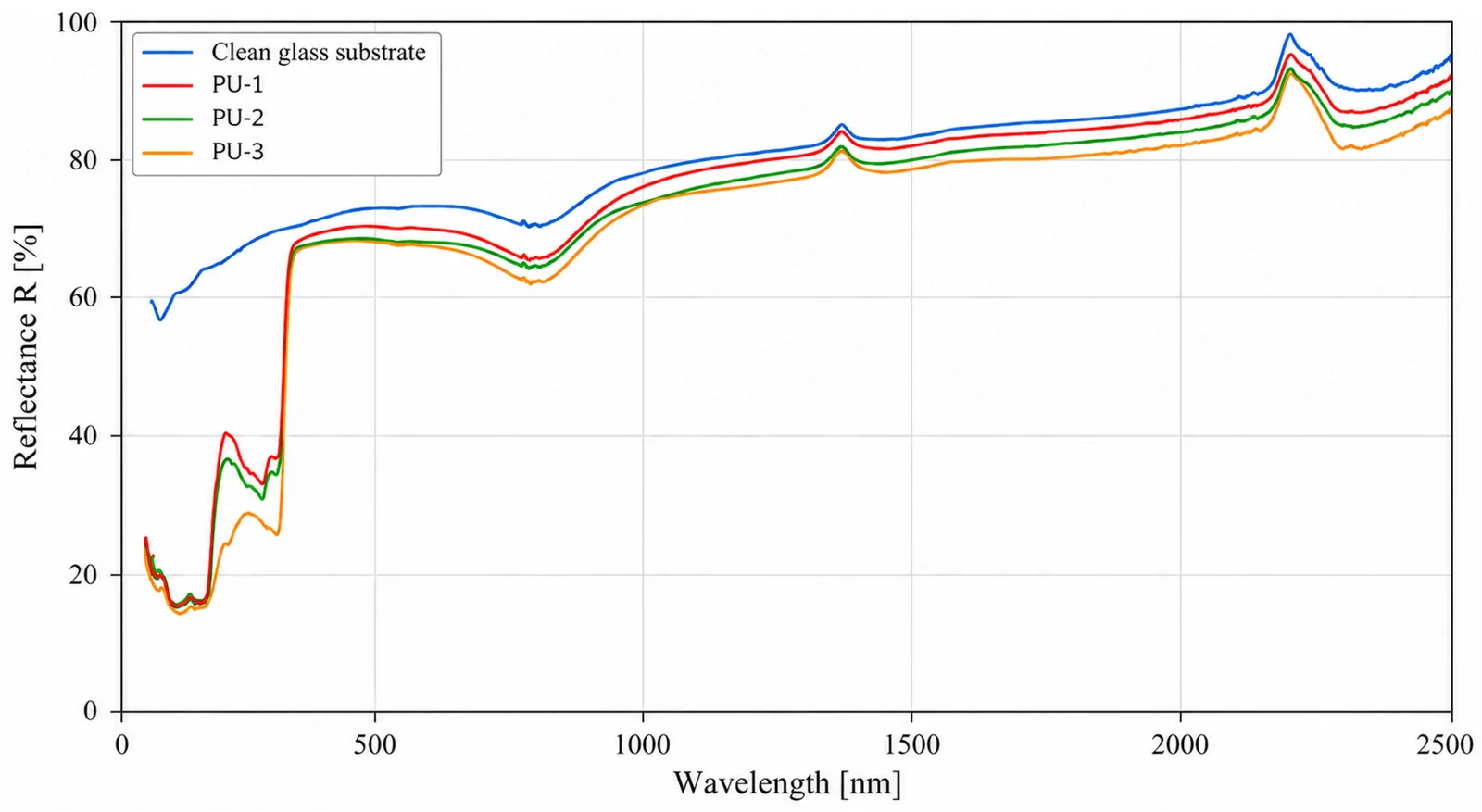
Comparison of reflectance spectra of the uncoated reference panel and polyurethane-coated specimens PU-1, PU-2 and PU-3.

**Figure 10:**
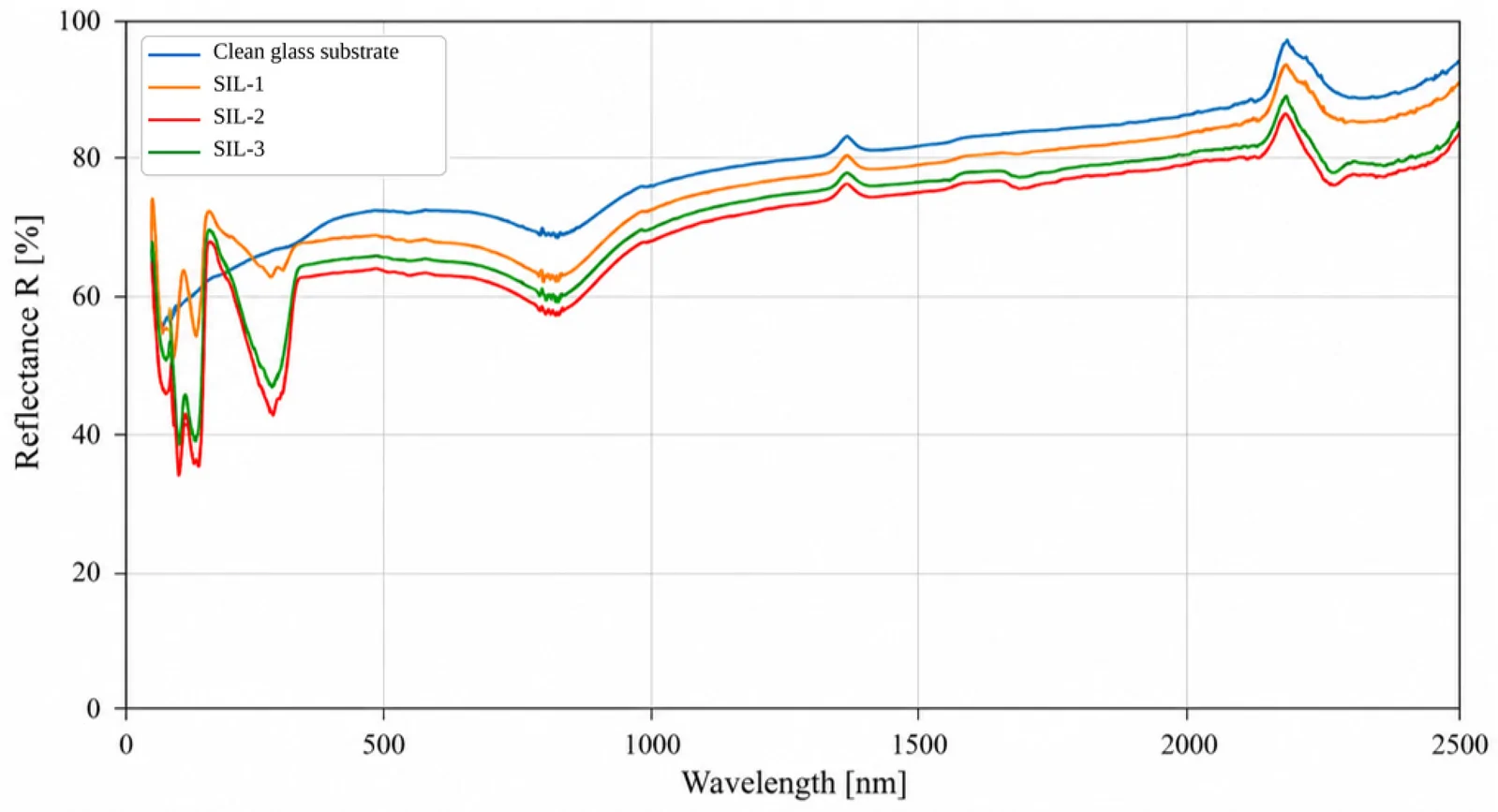
Comparison of reflectance spectra of the uncoated reference panel and silicone-modified coated specimens SIL-1, SIL-2 and SIL-3.

**Figure 11:**
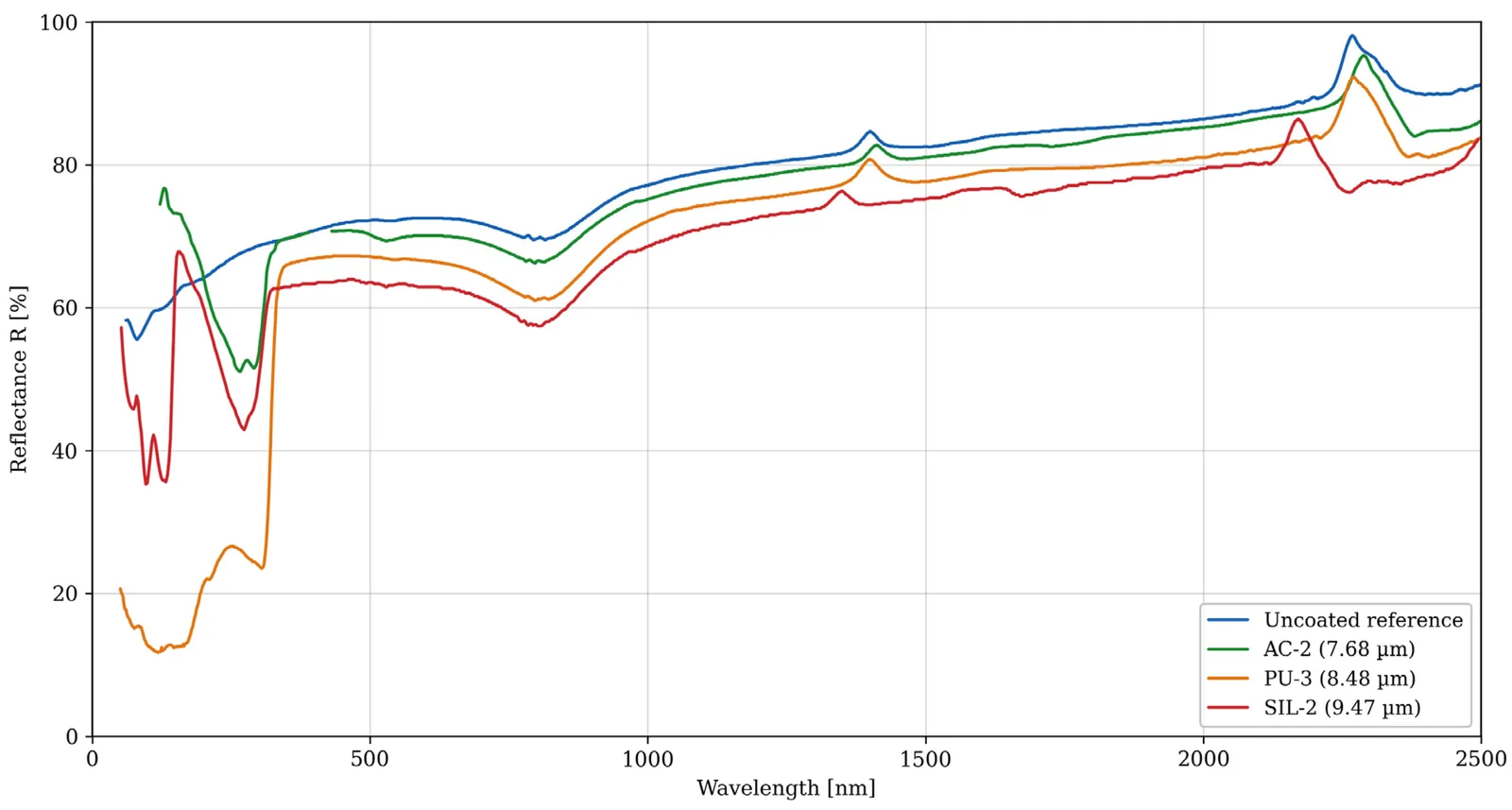
Comparison of reflectance spectra of the uncoated reference panel and specimens showing the similar dry-film thickness.

UV–Vis–NIR reflectance spectra (200–2500 nm) were compared for the uncoated reference and coatings prepared at nominal wet-film thicknesses of 30, 60, and 90 µm (Figure 7, Figure 8, Figure 9, Figure 10 and Figure 11).

The reference reflectance generally increased with wavelength and was highest in the NIR range (Figure 7). Acrylic coatings showed similar spectral profiles at all thicknesses, with reduced UV reflectance and high visible/NIR reflectance. The 60 µm variant gave the highest reflectance within this group (Figure 8).

Polyurethane coatings produced a larger reflectance reduction than acrylic coatings, especially at shorter wavelengths, while approaching the reference in the NIR range. The 30 µm variant showed the highest reflectance within this group (Figure 9).

Silicone-modified coatings differed only slightly with thickness and most closely retained the reference spectral profile; the 30 µm variant showed the highest reflectance over most of the range (Figure 10).

Comparison of the selected variants (Figure 11) confirmed that reflectance depends on coating type. The silicone-modified coating was closest to the uncoated reference, followed by acrylic, whereas polyurethane showed the largest reflectance reduction on 200–500 nm region among the compared variants. The UV–Vis–NIR reflectance spectra of coatings with similar experimentally measured dry-film thicknesses (AC-2: 7.68 µm, PU-3: 8.48 µm, and SIL-2: 9.47 µm) exhibited similar overall spectral profiles in Vis–NIR area, although differences in reflectance intensity were observed. Over most of the visible and near-infrared spectral ranges, the acrylic coating (AC-2) exhibited the highest reflectance among the coated specimens, followed by the polyurethane (PU-3) and silicone-modified acrylic (SIL-2) coatings, while the uncoated reference generally showed higher reflectance. These differences may be associated with the distinct chemical compositions of the investigated coatings; however, the small differences in dry-film thickness should also be considered when interpreting the results.

### 3.5. Thermal Properties

Thermal-property measurements showed that all investigated coatings affected heat transport through the substrate–coating system despite the small-layer thicknesses relative to the steel substrate. Changes in thermal conductivity λ, heat transfer coefficient U, and thermal resistance R were mutually consistent, i.e., an increase in λ corresponded to an increase in U and a decrease in R. Polymer type affected heat transport as strongly as the thickness of the coating applied to the steel substrate.

The analysis of changes in the thermal parameters of the systems did not reveal a single universal relationship between coating thickness and thermal properties; each of the investigated coatings exhibited its own characteristic behavior.

Table 2, Table 3 and Table 4 summarize the heat transfer coefficient, thermal resistance, and thermal conductivity values for the acrylic, polyurethane, and silicone-modified coatings, respectively.

**Figure 12:**
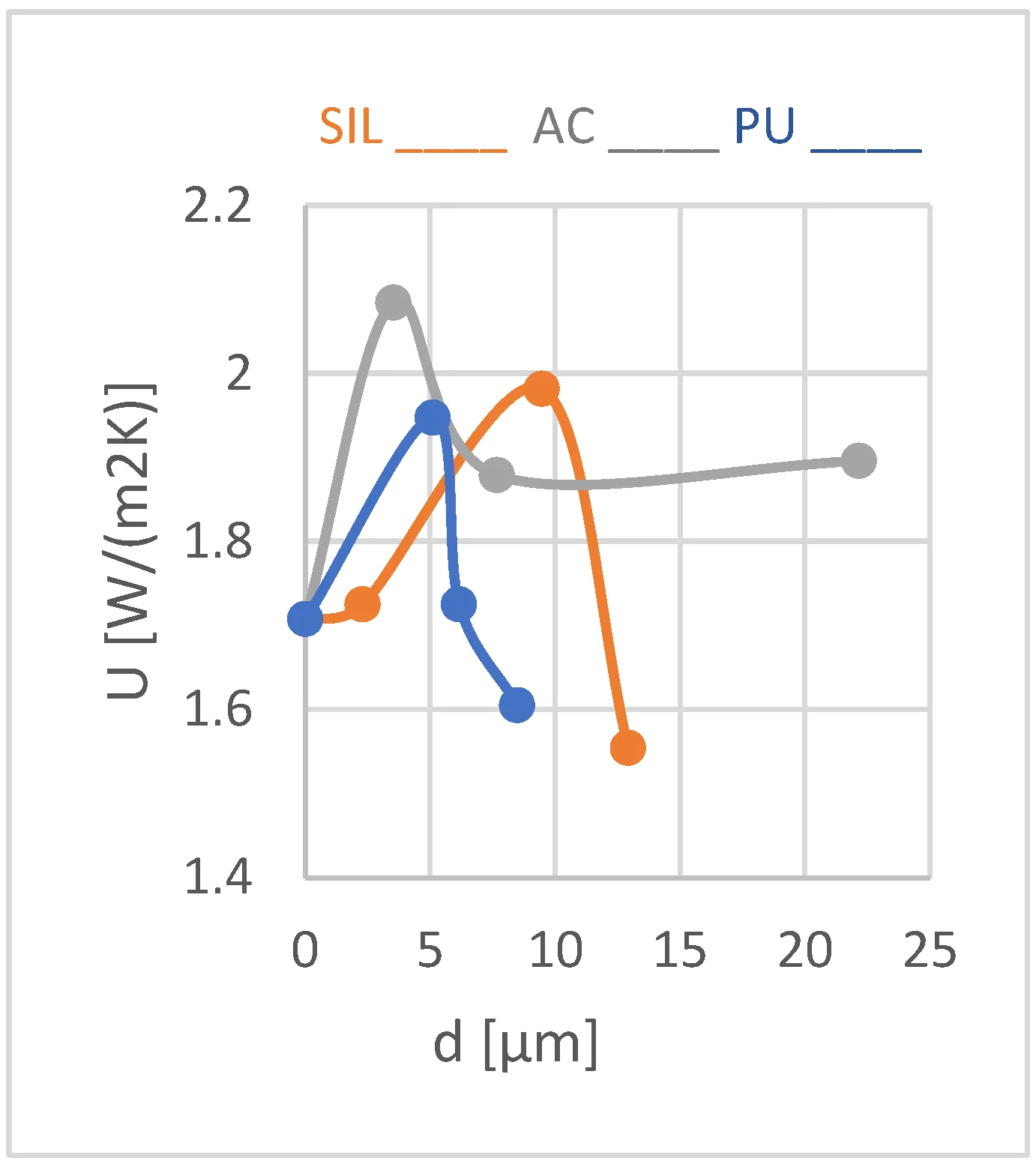
Heat transfer of the investigated steel specimens with protective coatings as a function of coating thickness.

**Figure 13:**
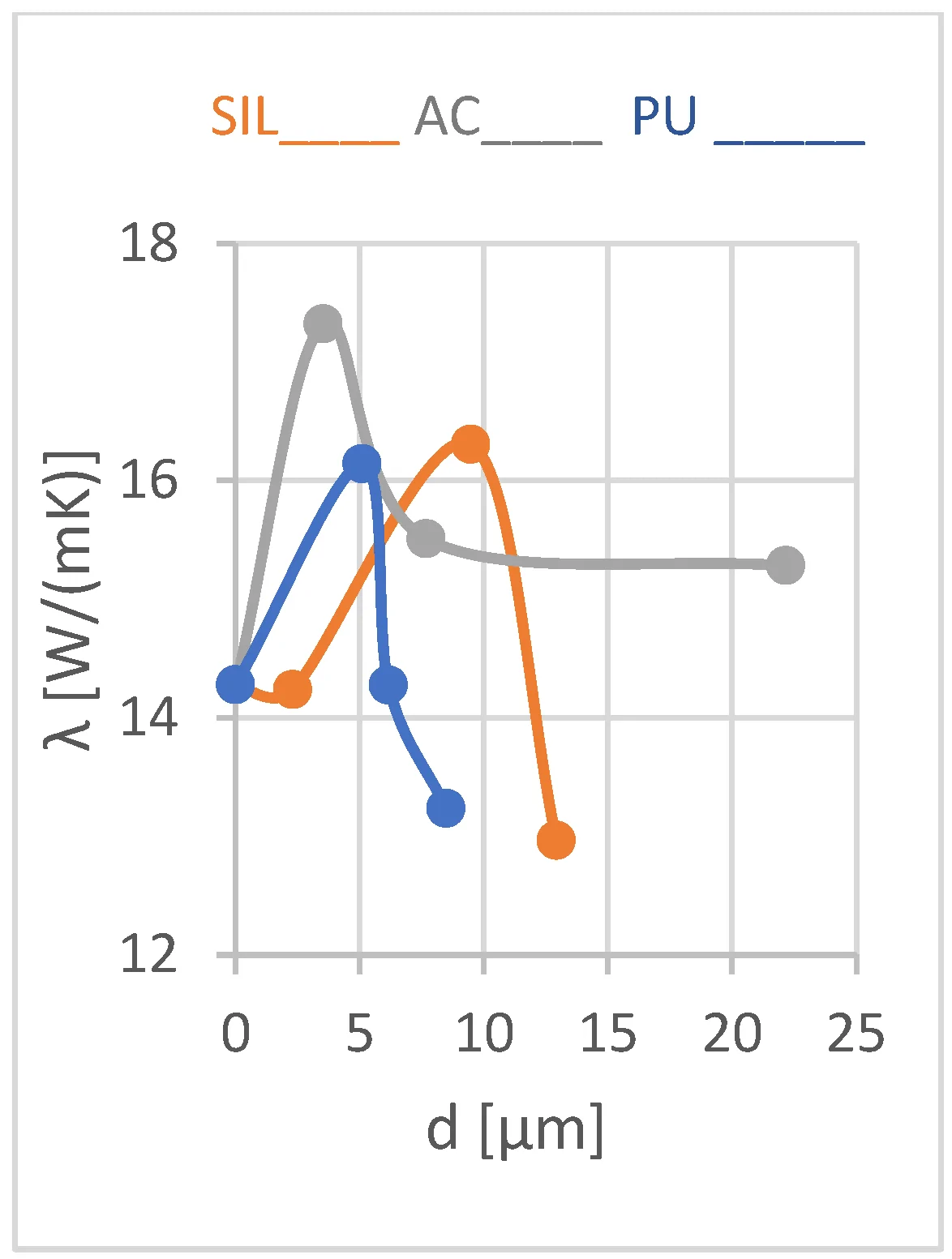
Thermal conductivity of the investigated steel specimens with protective coatings as a function of coating thickness.

**Figure 14:**
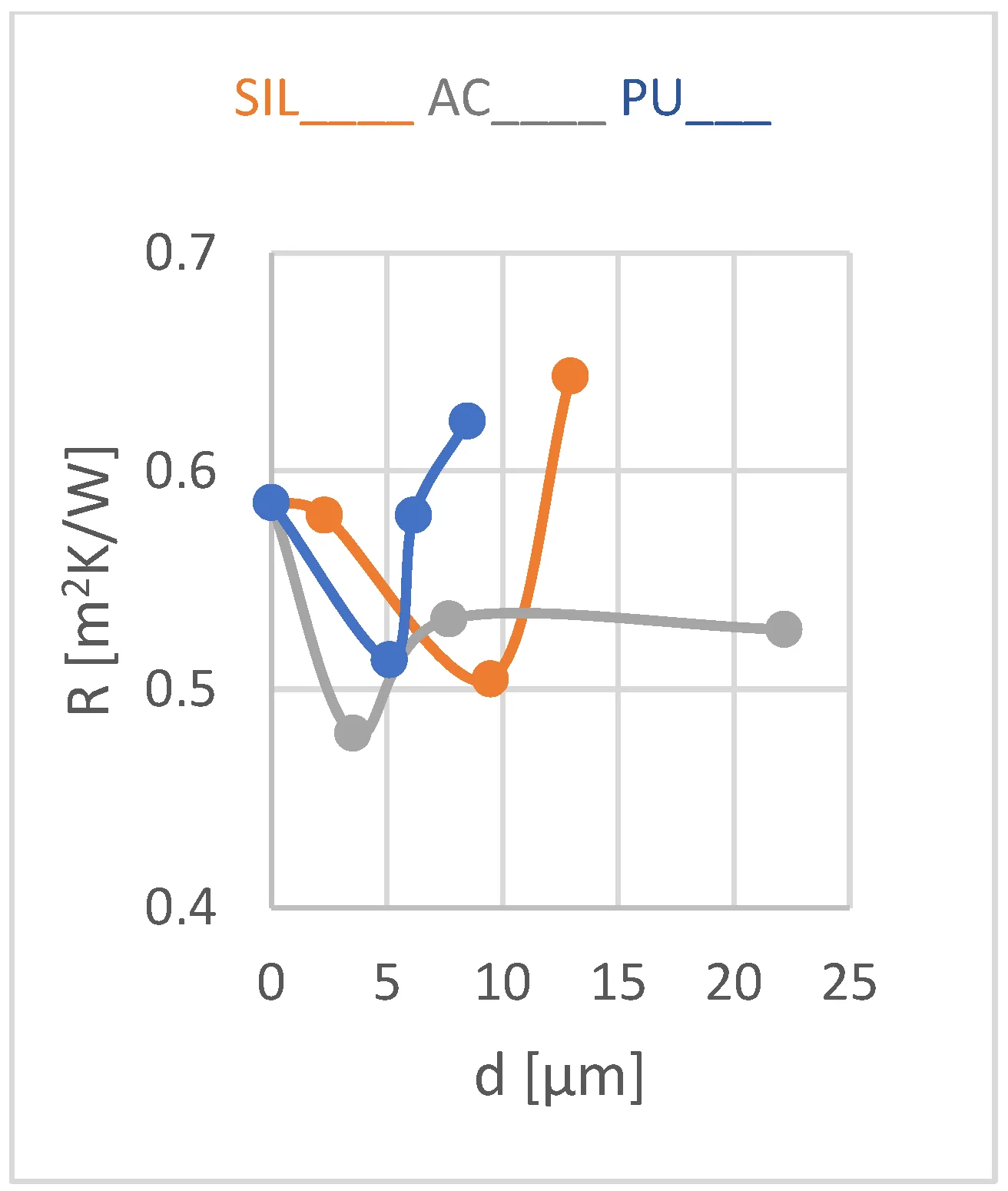
Thermal resistance of the investigated steel specimens with protective coatings as a function of coating thickness.

Figure 12, Figure 13 and Figure 14 show the heat transfer coefficient, thermal conductivity, and thermal resistance of the steel specimen with coating as a function of coating thickness for the individual coating types. The thermal contribution of the coating can typically be estimated when the heat transfer of the assembly exceeds that is of the substrate alone—that is when the coated assembly exhibits enhanced thermal conductivity compared to a reference steel plate.

Conversely, when the thermal conductivity or overall heat transfer coefficient of the coated assembly is lower than that of the reference steel plate, the coating acts as a thermal barrier. In such cases, the layer restricts heat flow through the substrate, which may be advantageous in applications requiring passive thermal protection or thermal isolation of sensitive electronic components. Polyurethane coatings (particularly PU-3) and silicone-modified coatings (SIL-3) demonstrated this thermal barrier behavior. In contrast, acrylic coatings maintained higher heat transfer capabilities across the investigated thickness range, facilitating heat conduction through the substrate.

**Figure 15:**
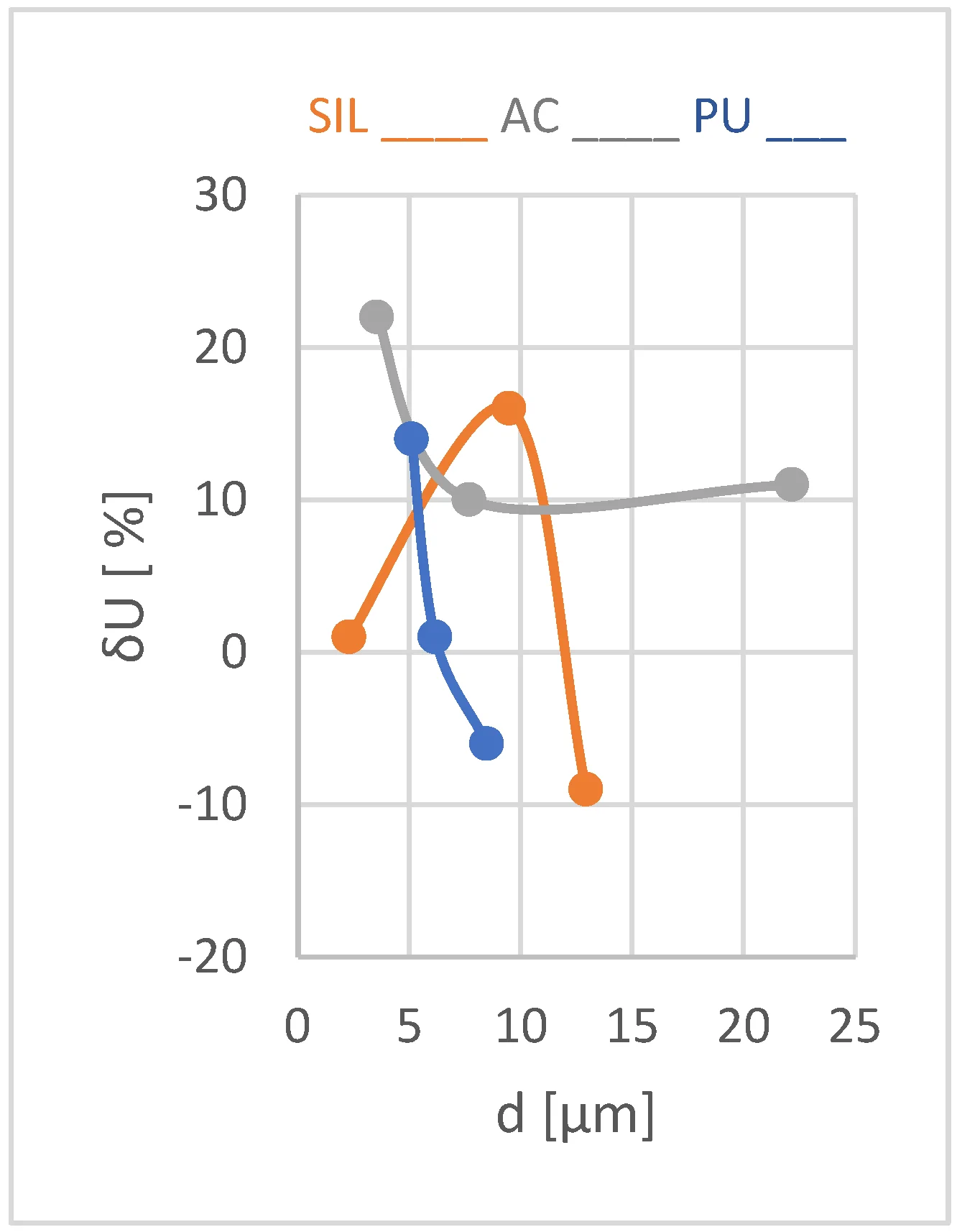
Change in heat transfer of the investigated protective coatings relative to an uncoated sample as a function of coating thickness.

**Figure 16:**
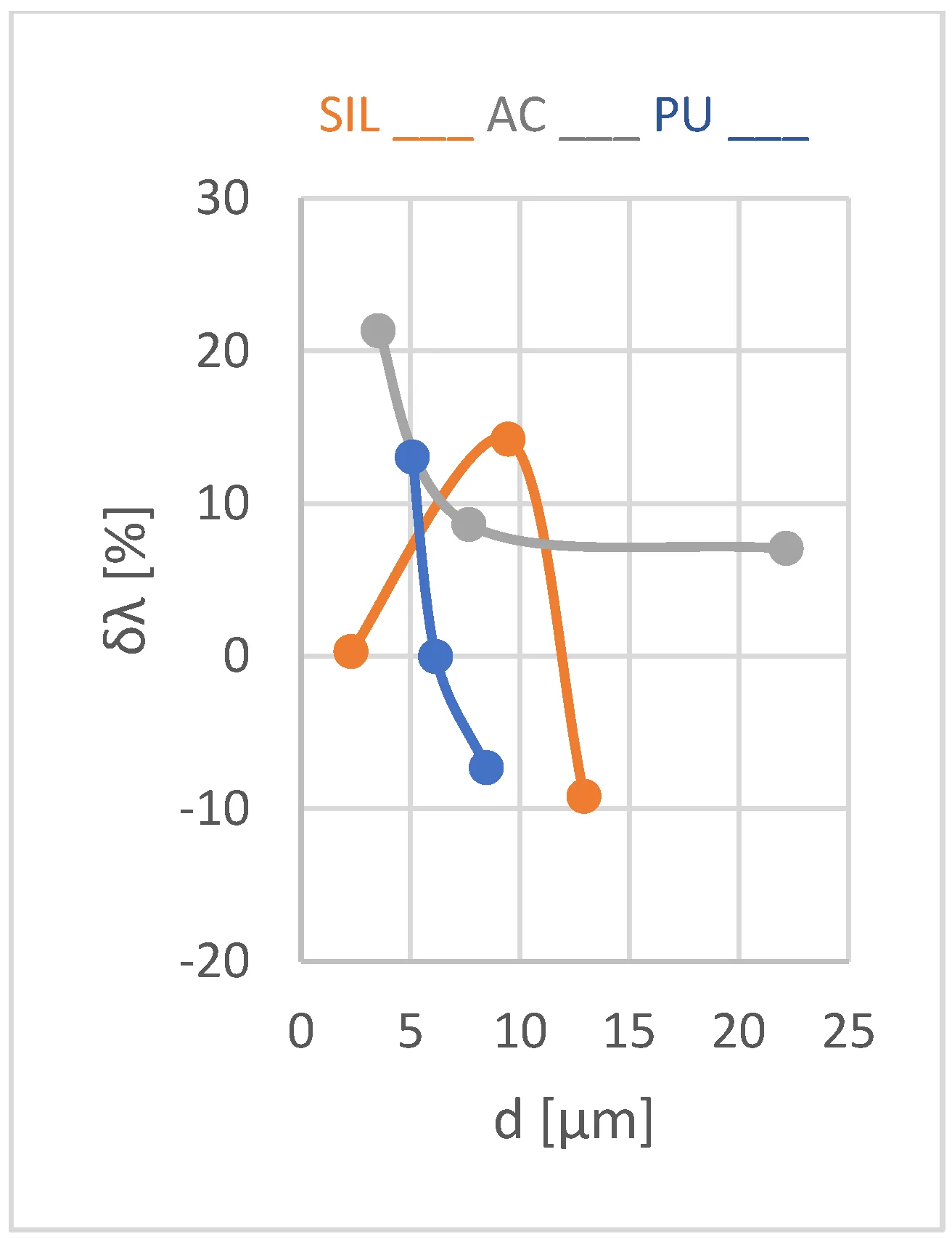
Change in thermal conductivity of the investigated protective coatings relative to an uncoated sample as a function of coating thickness.

**Figure 17:**
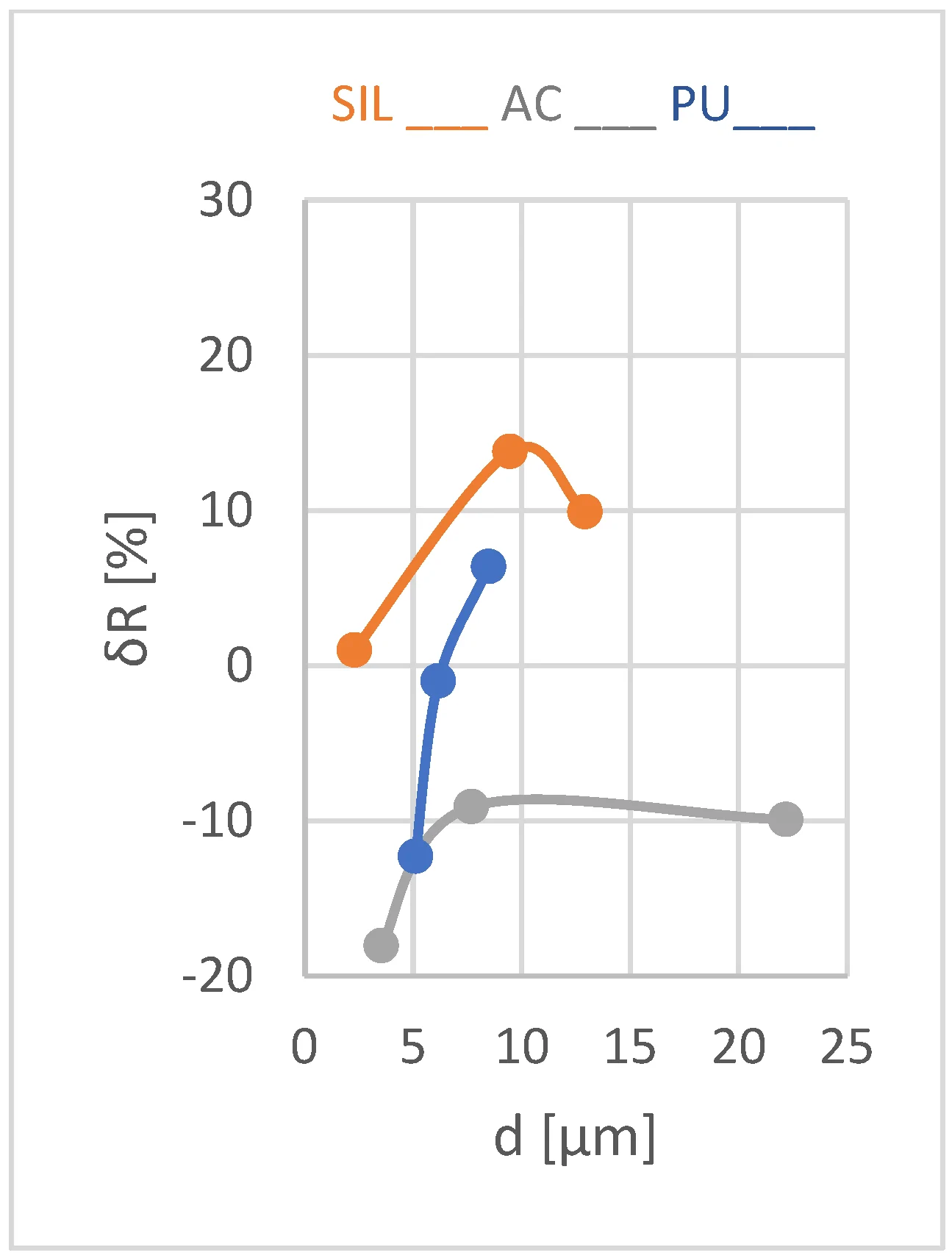
Change in thermal resistance of the investigated protective coatings relative to an uncoated sample as a function of coating thickness.

Figure 15, Figure 16 and Figure 17 show the relative changes in heat transfer coefficient, thermal conductivity, and thermal resistance with respect to the corresponding values of the uncoated reference specimen as a function of coating thickness for the individual types.

The acrylic coatings displayed relatively stable heat-conduction behavior. Positive changes in λ and U, along with negative changes in R, were generally maintained throughout the investigated range. The negative values of the relationship between thermal resistance and thickness (Figure 17) point to a substantial influence of the substrate on the apparent thermal performance. This material configuration appeared least sensitive to local thickness variations, which could be advantageous for electronic applications.

The silicone-modified coatings exhibited more complex behavior. As the dry-film thickness increased, heat transport initially seemed to improve, whereas at the greatest thickness, an increase in thermal resistance was observed. Based on the relative parameter changes as a function of coating thickness, it can be hypothesized that a thin layer may improve contact with the substrate, whereas at greater dimensions the intrinsic resistance of the coating itself begins to dominate.

Among the investigated formulations, the polyurethane layers exhibited the highest sensitivity to dry-film thickness variations. An increase in thickness resulted in a decrease in both thermal conductivity (λ) and the heat transfer coefficient (U), accompanied by a corresponding increase in thermal resistance (R). This strong thickness dependence underscores the critical need for precise control during the application process, especially in thermal management scenarios where efficient heat dissipation from a coated circuit board is essential.

Ultimately, these findings demonstrate that the binder type dictates how coating thickness governs heat transport. It should be emphasized, however, that the reported values reflect the effective thermal behavior of the composite substrate–coating assembly rather than the intrinsic bulk properties of standalone polymer films.

### 3.6. Surface Resistivity

All coatings exhibited very high surface resistivity, with values ranging from approximately 1.51–11.71 10^15^ Ω (Table 5), confirming their insulating character. The silicone-modified coating showed the highest values. Of all the samples tested, the silicone varnish had the highest surface resistivity values, demonstrating its greatest ability to effectively limit the flow of electric charges. Slightly lower surface resistivity values were recorded for the polyurethane and acrylic varnish samples. However, these parameters still remained at a high level, characteristic of professional protective coatings used in electronics.

The very high surface resistivity values obtained for all investigated coatings can be attributed to the intrinsically insulating nature of the polymeric matrices. Surface insulation resistance is an important parameter for assessing the electrical reliability of conformally coated electronic assemblies, since a high resistance limits leakage currents and reduces the possibility of electrically induced degradation between adjacent conductive elements. Previous studies have demonstrated that conformal coatings can significantly improve the surface insulation resistance and reliability of printed circuit boards, particularly when contamination and moisture are minimized [53]. The highest surface resistivity observed for the silicone-modified coating is consistent with the well-established electrical insulating properties of silicone-based materials. Silicone polymers are characterized by high surface resistivity, good dielectric properties, and resistance to environmental exposure, which explains their widespread use as insulating materials and conformal coatings in electrical and electronic applications [54,55]. Reported surface resistivity values for silicone coatings are of the same order of magnitude as those obtained in the present study. The slightly lower values obtained for the polyurethane and acrylic coatings can likewise be associated with differences in the chemical structure, greater polarity and moisture sensitivity. Nevertheless, both materials remain also highly insulating. For example, a surface resistivity of 6.99 × 10^14^ Ω has been reported for a polyurethane resin matrix [56], while acrylic polymer coatings used in electrical and electronic applications are generally classified as insulating materials when their surface resistivity exceeds 10^11^ Ω [57]. Therefore, the values measured for the investigated coatings, reaching the order of 10^15^ Ω, indicate a strong resistance to surface charge transport and confirm their suitability as electrically insulating protective coating materials. It should be noted, however, that surface resistivity is affected not only by the chemical nature of the polymer but also by coating uniformity, surface contamination, moisture, and measurement conditions. In particular, moisture and ionic contaminants can facilitate surface charge transport and cause a reduction in surface insulation resistance. However, the differences observed between the investigated coatings should be interpreted primarily as a result of their specific formulation and structure under the applied experimental conditions rather than as universal characteristics of silicone, polyurethane, and acrylic coatings [53].

In the context of automotive electronics, the differences in surface resistivity observed in this study may be relevant to the preliminary selection of conformal coatings for electrical insulation. The silicone-modified acrylic coating exhibited the highest surface resistivity under the applied laboratory conditions; however, this result alone does not establish its superior protective performance under elevated humidity or condensation. Previous studies on automotive electronic components have demonstrated the importance of coating integrity in limiting electrochemical migration [12]. Furthermore, experimental investigations have shown that coating adhesion, solder flux residues, and PCB surface cleanliness significantly affect the protective performance of conformal coatings under humid conditions [14,15,58]. Therefore, the present surface resistivity results should be interpreted as a comparative assessment of the electrical insulation properties of the investigated materials rather than evidence of their long-term reliability in automotive environments. Further testing on coated electronic assemblies under representative temperature, humidity, and electrical loading conditions would be required to evaluate their suitability for automotive applications.

### 3.7. Pendulum Hardness

König pendulum hardness measurements indicated differences associated with coating type and measured dry-film thickness. The reference-glass measurement gave a constant value of 170 oscillations, confirming correct instrument operation and measurement procedure. Detailed hardness results are presented in Table 6.

The obtained results indicate that the polyurethane and silicone coatings exhibited comparable average relative hardness, with values ranging from 0.71 to 0.73 in both cases, irrespective of coating thickness [59]. In contrast, the acrylic coatings showed a decrease in relative hardness with increasing thickness, which may be related to differences in the glass transition temperature of the coatings [60,61]. The differences between relative hardness the investigated coatings were therefore relatively small. The slightly lower hardness of the acrylic coating may result from the formation of a more flexible polymer network during the curing process. Furthermore, the mechanical properties of organic coatings are significantly influenced by the chemical structure of the binder, the degree of crosslinking, intermolecular interactions, and the resulting network structure. Previous studies have demonstrated that changes in crosslink density can significantly affect coating hardness and other mechanical properties [62]. In particular, an increase in crosslink density generally leads to higher hardness. The silicone coating exhibited the same relative hardness as the polyurethane coating (0.71–0.73). This result is consistent with literature reports indicating that silicone-based coatings can exhibit relatively high hardness, depending on their chemical structure and degree of crosslinking. At the same time, the mechanical properties of silicone coatings are strongly dependent on their composition; increasing the proportion of flexible silicone segments can significantly reduce coating hardness [62]. Therefore, the relatively high hardness of the silicone coating observed in this study may indicate a highly crosslinked and mechanically stable surface structure. Polyurethane coatings combine hardness with flexibility and impact resistance. Their final mechanical properties depend largely on the curing process, the content of soft and hard segments, the chemical structure, and the cross-linking density. The hardness of polyurethane coatings increases during curing as the polymer network develops [63].

## 4. Conclusions

The conducted study revealed clear differences in the chemical structure and functional properties of the investigated protective coatings. FTIR spectroscopy identified characteristic absorption bands consistent with the declared chemical composition of the investigated coatings. The acrylic coating exhibited bands associated with ester and aliphatic groups, the polyurethane coating showed characteristic aromatic and urethane-related bands, and the silicone-modified acrylic coating exhibited signals associated with both acrylic and siloxane structures. Optical microscopy further revealed differences in the size and distribution of the morphological structures observed within the coatings. UV–Vis–NIR spectrophotometry revealed differences in reflectance associated with coating type and thickness. For specimens with similar dry-film thicknesses (AC-2, PU-3, and SIL-2), the acrylic coating generally exhibited the highest reflectance among the coated specimens over most of the visible and near-infrared ranges. The results indicate that the optical response of the investigated coating–substrate systems depends on both the coating chemical composition and the thickness of the applied layer. Thermal analysis showed that all investigated coatings affected heat transport through the substrate–coating system. More favorable thermal properties were obtained for selected acrylic and silicone-modified acrylic coating variants, whereas the polyurethane coatings showed greater sensitivity of heat-transport parameters to changes in layer thickness. All investigated coatings exhibited high surface resistivity under the applied laboratory conditions. The highest mean value was recorded for the silicone-modified acrylic coating (11.71 × 10^15^ Ω), followed by polyurethane (4.79 × 10^15^ Ω) and acrylic (1.51 × 10^15^ Ω). These results indicate differences in electrical insulation properties between the investigated materials but do not establish their long-term electrical reliability under automotive operating conditions. König pendulum measurements showed comparable relative hardness values for the polyurethane and silicone-modified acrylic coatings, ranging from 0.71 to 0.73. In contrast, the relative hardness of the acrylic coating decreased from 0.73 to 0.62 with increasing dry-film thickness. The results indicate that the measured mechanical response depends on the coating formulation and, in the case of the acrylic coating, on film thickness. Overall, the results demonstrate that the investigated conformal coatings exhibit different combinations of functional properties, without identifying a single formulation that performs best across all evaluated parameters. The findings provide comparative laboratory data relevant to the preliminary selection of protective coatings for electronic applications. Therefore, selection of a coating for automotive electronics applications should simultaneously consider its structural, optical, thermal, electrical, and mechanical properties.

## Figures and Tables

**Table 1:** Experimentally measured dry-film thicknesses of the investigated coatings.

Sample Name	Mean Dry-Film Thickness [µm]
AC-1	3.53 ± 0.64
AC-2	7.68 ± 0.33
AC-3	22.17 ± 1.53
PU-1	5.10 ± 0.79
PU-2	6.15 ± 0.91
PU-3	8.48 ± 0.72
SIL-1	2.30 ± 0.37
SIL-2	9.47 ± 0.32
SIL-3	12.93 ± 0.12

**Table 2:** Thermal parameters of specimens coated with the acrylic coating and of the reference specimen.

Sample	Mean Dry-Film Thickness [µm]	Regression Slope Coefficient *a*_sh_, Mean ± u(*a*_sh_) [W/K]	Effective Thickness of the Sample–Holder System *d*_sh_ [m], u(*d*_sh_) = 0.00001 [m]	Heat Transfer Coefficient U ± u(U) [W/(m^2^·K)]	Thermal Conductivity λ ± u(λ) [W/(m·K)]	Thermal Resistance R ± u(R) [m^2^·K/W]
Uncoated reference steel plate	–	0.1010 ± 0.0005	0.00493	1.7 ± 0.01	14.2780 ± 0.008	0.585 ± 0.036
AC-1	3.53 ± 0.64	0.1032 ± 0.0008	0.00493	2.084 ± 0.078	17.321 ± 0.020	0.480 ± 0.037
AC-2	7.68 ± 0.33	0.1020 ± 0.0006	0.00493	1.879 ± 0.087	15.508 ± 0.021	0.532 ± 0.025
AC-3	22.17 ± 1.53	0.102 1 ± 0.0010	0.00493	1.896 ± 0.031	15.28 ± 0.013	0.5275 ± 0.0087

Values of *a*_sh_ represent the arithmetic mean of two independent measurement runs (n = 2), with the reported uncertainty corresponding to the maximum absolute deviation from the mean.

**Table 3:** Thermal parameters of specimens coated with the polyurethane coating and of the reference specimen.

Sample	Mean Dry-Film Thickness [µm]	Regression Slope Coefficient *a*_sh_, Mean ± u(*a*_sh_) [W/K]	Effective Thickness of the Sample–Holder System *d*_sh_ [m], u(d*_sh_*) = 0.00001 [m]	Heat Transfer Coefficient U ± u(U) [W/(m^2^·K)]	Thermal Conductivity λ ± u(λ) [W/(m·K)]	Thermal Resistance R ± u(R) [m^2^·K/W]
Uncoated reference steel plate	–	0.1010 ± 0.0005	0.00493	1.7 ± 0.01	14.278 ± 0.008	0.585 ± 0.036
PU-1	5.10 ± 0.79	0.1024 ± 0.0010	0.00493	1.95 ± 0.20	16.141 ± 0.032	0.51 ± 0.07
PU-2	6.15 ± 0.91	0.1011 ± 0.0008	0.00493	1.72 ± 0.21	14.274 ± 0.033	0.579 ± 0.072
PU-3	8.48 ± 0.72	0.1004 ± 0.0006	0.00493	1.605 ± 0.15	13.233 ± 0.028	0.62 ± 0.06

**Table 4:** Thermal parameters of specimens coated with the silicone-modified coating and of the reference specimen.

Sample	Mean Dry-Film Thickness [µm]	Regression Slope Coefficient *a*_sh_, Mean ± u(*a*_sh_) [W/K]	Effective Thickness of the Sample–Holder System *d*_sh_ [m], u(d*_sh_*) = 0.00001 [m]	Heat Transfer Coefficient U ± u(U) [W/(m^2^·K)]	Thermal Conductivity λ ± u(λ) [W/(m·K)]	Thermal Resistance R ± u(R) [m^2^·K/W]
Uncoated reference steel plate	–	0.1010 ± 0.0005	0.00493	1.7 ± 0.01	14.278 ± 0.008	0.585 ± 0.036
SIL-1	2.30 ± 0.37	0.1013 ± 0.0008	0.00493	1.72 ± 0.17	14.235 ± 0.030	0.5797 ± 0.0035
SIL-2	9.47 ± 0.32	0.1026 ± 0.0012	0.00493	1.98 ± 0.32	16.306 ± 0.040	0.505 ± 0.083
SIL-3	12.93 ± 0.12	0.1001 ± 0.006	0.00493	1.55 ± 0,14	12.966 ± 0.027	0.64 ± 0.06

**Table 5:** Surface resistivity of investigated coatings.

Sample	Sample 1	Sample 2	Mean Surface Resistivity
AC	(1.47 ± 0.01) × 10^15^ Ω	(1.55 ± 0.02) × 10^15^ Ω	(1.51 ± 0.23) × 10^15^ Ω
PU	(4.71 ± 0.05) × 10^15^ Ω	(4.87 ± 0.05) × 10^15^ Ω	(4.79 ± 1.30) × 10^15^ Ω
SIL	(12.19 ± 0.12) × 10^15^ Ω	(11.23 ± 0.11) × 10^15^ Ω	(11.71 ± 7.18) × 10^15^ Ω

**Table 6:** Results of König pendulum hardness measurements.

Sample	Mean Dry-Film Thickness [µm]	Average Number of Oscillations	Average Relative Hardness
Reference glass	–	170.0 ± 0.0	-
AC-1	3.53 ± 0.64	124.3 ± 1.5	0.73 ± 0.03
AC-2	7.68 ± 0.33	118.7 ± 2.1	0.70 ± 0.03
AC-3	22.17 ± 1.53	105.0 ± 2.6	0.62 ± 0.05
PU-1	5.10 ± 0.79	122.0 ± 4.4	0.72 ± 0.03
PU-2	6.15 ± 0.91	121.0 ± 3.0	0.71 ± 0.02
PU-3	8.48 ± 0.72	124.0 ± 1.0	0.73± 0.02
SIL-1	2.30 ± 0.37	124.3 ± 3.1	0.73 ± 0.01
SIL-2	9.47 ± 0.32	121.3 ± 3.1	0.71 ± 0.02
SIL-3	12.93 ± 0.12	123.3 ± 2.1	0.73 ± 0.01

## Data Availability

The original contributions presented in this study are included in the article. Further inquiries can be directed to the corresponding authors.
